# Genetic analysis and molecular basis of G6PD deficiency among malaria patients in Thailand: implications for safe use of 8-aminoquinolines

**DOI:** 10.1186/s12936-024-04864-8

**Published:** 2024-02-02

**Authors:** Usa Boonyuen, Beatriz Aira C. Jacob, Jutamas Wongwigkan, Kamonwan Chamchoy, Natsamon Singha-art, Natnicha Pengsuk, Duantida Songdej, Emily R. Adams, Thomas Edwards, Supat Chamnanchanunt, Syazwani Itri Amran, Nurriza Ab Latif, Naveen Eugene Louis, Shamini Chandran

**Affiliations:** 1 Department of Molecular Tropical Medicine and Genetics, Faculty of Tropical Medicine, Mahidol University, Bangkok, Thailand; 2 Princess Srisavangavadhana College of Medicine, Chulabhorn Royal Academy, Bangkok, Thailand; 3 Department of Pediatrics, Faculty of Medicine Ramathibodi Hospital, Mahidol University, Bangkok, Thailand; 4 Centre for Drugs and Diagnostics Research, Liverpool School of Tropical Medicine, Liverpool, UK; 5 Department of Clinical Tropical Medicine, Faculty of Tropical Medicine, Mahidol University, Bangkok, Thailand; 6 Department of Bioscience, Faculty of Science, Universiti Teknologi Malaysia, Johor Bahru, Malaysia

**Keywords:** G6PD deficiency, Malaria, *G6PD* mutations, *G6PD* genotyping, Synonymous mutations, Stability

## Abstract

**Background:**

It was hypothesized that glucose-6-phosphate dehydrogenase (G6PD) deficiency confers a protective effect against malaria infection, however, safety concerns have been raised regarding haemolytic toxicity caused by radical cure with 8-aminoquinolines in G6PD-deficient individuals. Malaria elimination and control are also complicated by the high prevalence of G6PD deficiency in malaria-endemic areas. Hence, accurate identification of G6PD deficiency is required to identify those who are eligible for malaria treatment using 8-aminoquinolines.

**Methods:**

The prevalence of G6PD deficiency among 408 Thai participants diagnosed with malaria by microscopy (71), and malaria-negative controls (337), was assessed using a phenotypic test based on water-soluble tetrazolium salts. High-resolution melting (HRM) curve analysis was developed from a previous study to enable the detection of 15 common missense, synonymous and intronic *G6PD* mutations in Asian populations. The identified mutations were subjected to biochemical and structural characterisation to understand the molecular mechanisms underlying enzyme deficiency.

**Results:**

Based on phenotypic testing, the prevalence of G6PD deficiency (< 30% activity) was 6.13% (25/408) and intermediate deficiency (30–70% activity) was found in 15.20% (62/408) of participants. Several *G6PD* genotypes with newly discovered double missense variants were identified by HRM assays, including *G6PD* Gaohe + Viangchan, *G6PD* Valladolid + Viangchan and *G6PD* Canton + Viangchan. A significantly high frequency of synonymous (c.1311C>T) and intronic (c.1365-13T>C and c.486-34delT) mutations was detected with intermediate to normal enzyme activity. The double missense mutations were less catalytically active than their corresponding single missense mutations, resulting in severe enzyme deficiency. While the mutations had a minor effect on binding affinity, structural instability was a key contributor to the enzyme deficiency observed in G6PD-deficient individuals.

**Conclusions:**

With varying degrees of enzyme deficiency, *G6PD* genotyping can be used as a complement to phenotypic screening to identify those who are eligible for 8-aminoquinolines. The information gained from this study could be useful for management and treatment of malaria, as well as for the prevention of unanticipated reactions to certain medications and foods in the studied population.

**Supplementary Information:**

The online version contains supplementary material available at 10.1186/s12936-024-04864-8.

## Background

Although there is a substantial decrease in global morbidity and mortality attributed to malaria, an estimate of 247 million cases were reported by the World Health Organization (WHO) in 2021, with approximately 619,000 deaths worldwide [1]. Moreover, in countries striving for malaria eradication, greater focus is directed towards *Plasmodium vivax* and *Plasmodium ovale* parasites, which have dormant liver stage forms that can lead to relapse. The only class of medications utilised for preventing relapse and achieving radical cure of malaria is 8-aminoquinolines, namely primaquine and tafenoquine. However, the administration of these anti-malarial drugs in individuals with glucose-6-phosphate dehydrogenase (G6PD) deficiency can cause serious side effects, drawing safety concerns. Thus, the WHO acknowledges that two diagnoses are required towards the safe and effective radical treatment of malaria: the presence of *P. vivax* parasites and G6PD deficiency status [2].

G6PD deficiency is the most common human erythro-enzymopathy caused by inherited mutations in the *G6PD* gene [3, 4]. The G6PD enzyme catalyses the initial step of the pentose phosphate pathway, generating reduced nicotinamide adenine dinucleotide phosphate (NADPH) required in cellular oxidative defense mechanisms [5]. G6PD with reduced enzyme activity is, therefore, unable to provide NADPH at a normal rate in erythrocytes, increasing susceptibility to haemolysis during oxidative challenge [6]. Nearly all G6PD-deficient individuals do not exhibit signs and symptoms, unless induced by an exogenous source of oxidative stress such as 8-aminoquinolines, causing acute haemolytic anaemia characterised by jaundice, haemoglobinuria, and flank pain [7, 8]. It has also been proposed that G6PD deficiency confers a relative protection against severe malaria, as reflected by the geographic frequency overlap between the two [9–11]. However, an established mechanism of protection has yet to be described, as well as the extent in allele-carrying individuals, especially in hemizygous males and heterozygous females [12–15]. Despite this protective effect, malaria therapeutics are still complicated by drug-induced haemolysis in G6PD-deficient individuals. Quantitative and qualitative techniques to diagnose G6PD deficiency have been developed over time, but these are limited by risks of misclassification and other haematological parameters [16]. To overcome these limitations, genetic testing is currently being advanced to reliably identify the G6PD deficiency status of a patient [17].

*G6PD* mutations are associated with various degrees of enzymatic activity and haemolytic vulnerability. There are now over 230 *G6PD* variants with known mutations, which are prominently missense mutations or small in-frame deletions [18]. These alleles have been classified based on the level of G6PD activity in erythrocytes and the clinical manifestations of the allele-carrying individuals [19]. Even though most *G6PD* variants are identified as single point mutations, multiple missense and intronic mutations causative of G6PD deficiency have been increasingly identified as well [20, 21].

In Thailand, 18,949 cases of malaria have been reported from January 2022 to June 2023, with *P. vivax* and *P. ovale* accounting for 94% of infections (malaria.ddc.moph.go.th). This implies the importance of utilising 8-aminoquinolines towards malaria elimination in the country. Moreover, the prevalence of G6PD deficiency in Thailand lies in the range of 3 to 18%, depending on ethnicity and geographical location, with more than 20 variants identified [22–26]. The most common single mutations, *G6PD* Viangchan (c.871G>A, p.Val291Met) and *G6PD* Mahidol (c.487G>A, p.Gly163Ser), can also give rise to individuals carrying the double mutant G6PD Mahidol + Viangchan with reduced catalytic efficiency and protein instability compared to the single mutations [27, 28].

In this study, a quantitative phenotypic test for G6PD deficiency was conducted among Thai malaria patients and malaria-negative controls. *G6PD* genotyping was carried out via multiplex high-resolution melting (HRM) assay [29], which has been further developed to detect synonymous and intronic mutations common in Thai people (c.1311C>T, c.1365-13C>T, and c.486-34delT). To better understand the effect of single and double missense mutations on G6PD activity, functional and structural analyses of variants identified by genotyping, namely, G6PD Gond (p.Met159Ile), G6PD Gaohe + Viangchan (p.His27Arg + Val298Met), G6PD Valladolid + Viangchan (p.Arg136Cys + Val298Met) and G6PD Canton + Viangchan (p.Arg459Leu + Val298Met), were performed and compared to their corresponding single variants. Moreover, computational analysis was carried out to elucidate the structural changes of G6PD variants in order to provide insights into the structure‒function relationship.

## Methods

### Ethics

The study was approved by the Human Ethics Committee of the Faculty of Tropical Medicine, Mahidol University (approval number MUTM 2021-075-02). The participants provided written consent to have their specimens used in the research.

### Blood samples

**Fig. 1:**
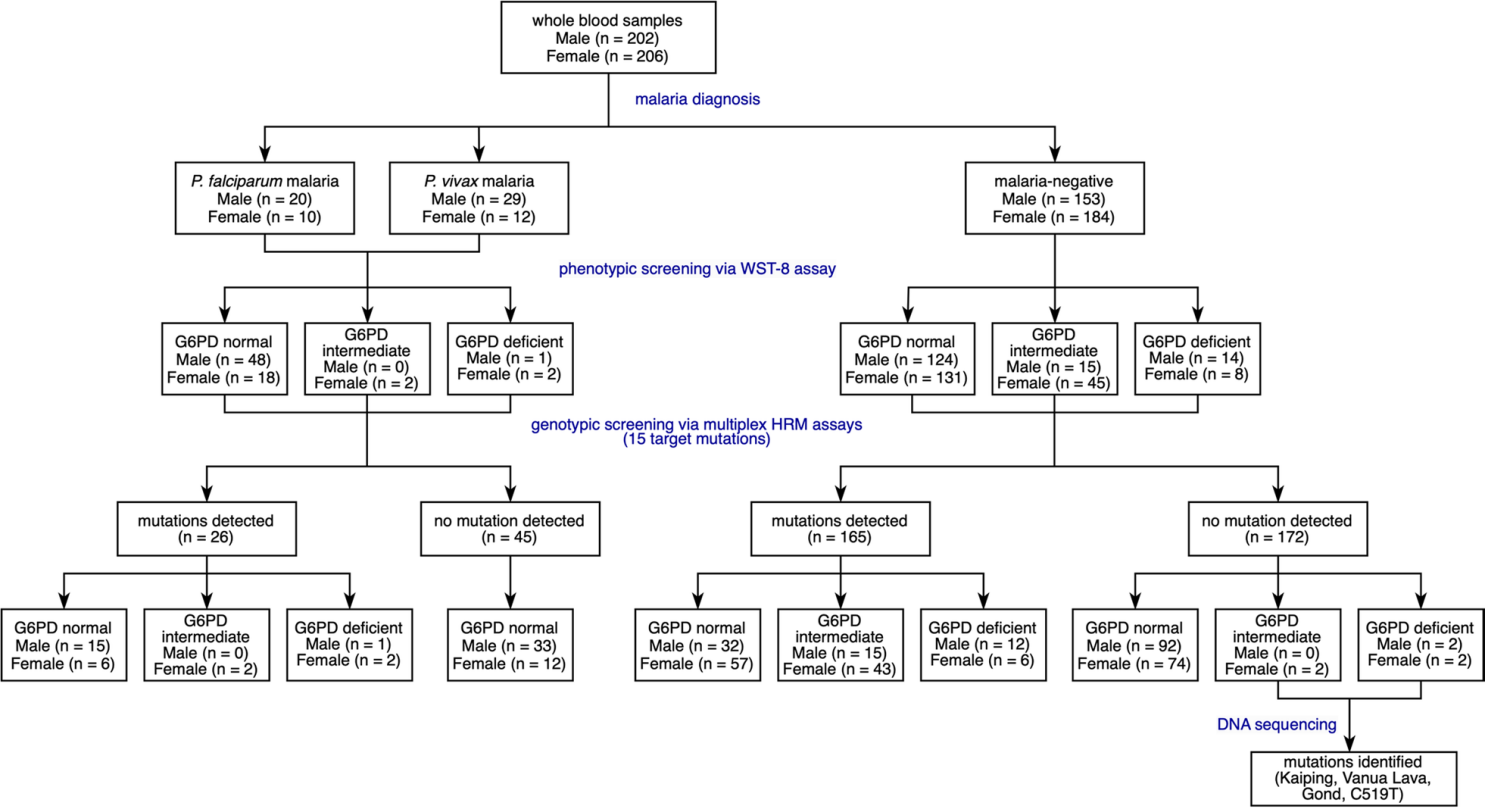
Study flow chart

This retrospective study was carried out using archived blood samples collected at the Hospital for Tropical Diseases, Bangkok, Thailand during 2013–2019. Blood samples were stored at ‒20 °C until use. Under this storage condition, the integrity of samples for phenotypic screening was maintained [30]. The data were fully anonymised and the authors had no access to information that could identify individual participants. The study design is depicted in Fig. 1.

### Malaria detection

*Plasmodium* infection was diagnosed by two independent well-trained microscopists via analysis of Giemsa-stained thin blood smears. Identification of the *Plasmodium* species was performed using polymerase chain reaction (PCR)-based protocols [31].

### Phenotypic characterisation of G6PD deficiency using WST-8 assay

The prevalence of G6PD deficiency was determined by a phenotypic test based on water-soluble tetrazolium salts (WST-8) assay, following prior study’s protocols [32]. The activity of G6PD was expressed as units (U) per gram of haemoglobin (Hb).

### Genomic DNA extraction

Genomic DNA extraction was performed using a QIAamp DNA Blood Mini Kit (QIAGEN, Hilden, Germany), according to the manufacturer’s instructions. A 100 μL of each blood sample was mixed with 100 μL phosphate buffer saline, extracted and eluted into a final volume of 100 μL. The DNA concentration was determined using a NanoDrop 2000 spectrophotometer (Thermo Fisher Scientific, Waltham, MA, USA).

### G6PD genotyping by HRM assays

Previously, multiplex HRM assays that can detect 12 mutations common in Thailand and Southeast Asia in three reactions were reported [29]. Nonetheless, three further *G6PD* genotypes are highly prevalent in the Asian population, including a synonymous mutation in exon 11 (c.1311C>T), an intron 11 mutation (c.1365-13T>C) and a deletion in intron 5 (c.486-34delT) [33–35]. Hence, in this study, the HRM assays to enable the detection of these three mutations were further developed (Additional file 1: Fig. S1). Primers were designed flanking each target point mutation in the *G6PD* gene, to generate amplicons with different melting temperatures (T_m_) depending on the presence of the mutation, Additional file 1: Table S1.

Multiplex HRM experiments were set up and performed in accordance with previously published protocols [29]. The performance of HRM assays was assessed. The number of true positives (TP), true negatives (TN), false positives (FP), and false negatives (FN) were determined with DNA sequencing as a reference method. A total of 175 blood samples with known *G6PD* genotypes (100 *G6PD*-mutant and 75 *G6PD*-wild type (WT)) were used to determine the specificity and sensitivity of the assay. The HRM assays were then used to screen 408 Thai participants for *G6PD* mutations.

### DNA sequencing

Samples with impaired G6PD activity but no mutations detected by HRM assays (Fig. 1) were amplified for the *G6PD* gene and sent for sequencing. PCR amplification was carried out following a published methodology, covering exons 2 to 13 and introns 3, 4, 6, 7, and 9‒12 [32], and PCR products were purified and sequenced commercially (1st BASE; Apical Scientific, Selangor, Malaysia).

### Biochemical and structural characterisation of G6PD variants

*G6PD* genotyping identified various *G6PD* variants among the studied population, including *G6PD* Gond, *G6PD* Gaohe + Viangchan, *G6PD* Valladolid + Viangchan and *G6PD* Canton + Viangchan. The presence of these mutations was verified by Sanger sequencing. Both HRM assays and Sanger sequencing cannot determine whether the double mutations are in the *cis* or *trans* configuration. To understand the molecular mechanisms underlying enzyme deficiency of these four variants, biochemical and structural characterisation was carried out. To assess the combined effects of double mutations, they were created as in *cis* configuration and their biochemical and structural properties were compared with WT and corresponding single mutations.

#### Site-directed mutagenesis and protein expression and purification

Site-directed mutagenesis was carried out to create *G6PD* variants. Single missense mutations were constructed using the pET28a-*G6PD* WT template while double mutations were created using the pET28a-*G6PD* Viangchan template. Primers used for site-directed mutagenesis are listed in Additional file 1: Table S2. The PCR conditions for site-directed mutagenesis were previously described [36]. The presence of desired mutations was confirmed by DNA sequencing.

G6PD protein was expressed in *Escherichia coli* BL21 (DE3) and purified to homogeneity using immobilised metal affinity chromatography in accordance with the previously described protocols [29]. Protein purity was visualised with sodium dodecyl sulfate–polyacrylamide gel electrophoresis and the protein concentration was determined by the Bradford assay [37].

#### Determination of steady-state kinetic parameters of G6PD variants

Steady-state kinetic parameters were determined to assess the effect of mutations on the catalytic activity of G6PD variants. Experiments were carried out following previous report [29]. To determine the *K*_m_ for glucose-6-phosphate (G6P), the concentration of oxidized nicotinamide adenine dinucleotide phosphate (NADP^+^) was fixed at 100 μM while varying the concentrations of G6P from 2.5 to 1000 µM and to determine the *K*_m_ for NADP^+^, the concentration of G6P was fixed at 500 μM while varying the concentrations of NADP^+^ from 1 to 200 µM.

#### Structural characterisation of G6PD variants

Structural analyses of G6PD variants were performed according to previous reports [29, 36]. The secondary structure of G6PD proteins was analysed using circular dichroism (CD) to determine the effect of mutations on the secondary structure of G6PD variants. Far UV-CD spectra of the G6PD variants (0.1 mg/mL) were recorded in a 1 mm path-length quartz cuvette at 25 °C using a Jasco spectrometer, model J-815, equipped with a Peltier temperature control system.

Thermal stability analysis was performed in a 20 μL reaction, containing protein at a concentration of 0.25 mg/mL mixed with 5 × SYPRO Orange Protein Gel Stain (Thermo Fisher Scientific, San Jose, CA, USA). The reaction mixtures were heated in a Light‒Cycler 480 real-time PCR machine (Roche, Mannheim, Germany) at temperatures ranging from 20 to 80 °C, with excitation and emission wavelengths of 465 and 580 nm, respectively. Furthermore, the effect of NADP^+^ was investigated by incubating the protein in the presence of various concentrations of NADP^+^ (0, 10 and 100 µM). The melting temperature (*T*_*m*_) of each G6PD variant was calculated and defined as the temperature at which half of the protein was unfolded.

To assess the effect of mutations on the structural stability of G6PD variants, the enzyme was incubated for 20 min at temperatures ranging from 25 to 65 °C in the presence of various concentrations of NADP^+^ (0, 10 and 100 μM) before being cooled to 4 °C in a Thermocycler (Eppendorf, Hamburg, Germany). The residual enzyme activity was measured and expressed as a percentage of the activity of the same enzyme incubated at 25 °C.

To investigate the structural stability of G6PD variants upon chemical denaturation, the protein was treated with different concentrations of guanidine hydrochloride (Gdn-HCl; 0 to 0.5 M) in the presence of various concentrations of NADP^+^ (0, 10 and 100 µM) at 37 °C for 2 h. The residual enzyme activity was measured and expressed as a percentage of the activity of the same enzyme incubated without Gdn-HCl.

To determine the susceptibility of G6PD variants to trypsin digestion, the protein was treated with trypsin (0.5 mg/mL) for 5 min at 25 °C in the presence of various concentrations of NADP^+^ (0, 10 and 100 µM). The residual enzyme activity was measured and expressed as a percentage of the activity of the same enzyme incubated without trypsin.

### Molecular docking and molecular dynamic simulation (MDS)

Molecular docking was performed to construct the G6PD dimeric complex with G6P and NADP^+^ ligands retrieved from PDB ID:2BHL and 2BH9 [38] using AutoDock 4.2 software.* In silico* site-directed mutagenesis was performed using the WT structure to construct the mutant enzymes using mutagenesis tool in the PyMOL software (PyMOL Molecular Graphics System, Schrödinger, LLC).

The WT and prepared mutants were subjected for simulation using the GROMACS 2018.1 package. The pdb2gmx utility and the GROMOS96 54a7 force field were utilised for protein preparation while ligand topology files were prepared using the Automated Topology Builder [39, 40]. The protein–ligand complex of the WT and mutants was assembled by merging the topology and atomic coordinates of the protein and ligands. The system was solvated using a simple point charge water box and then neutralised by adding counter Na^+^ ions before the energy was minimised at 50,000 steps using the steepest descent method. The system was subjected to 5000 steps of constant number of particles, volume, and temperature for 100 ps at 300 K in the equilibration step. The constraint and electrostatic interactions were established by employing the linear constraint solver and the particle mesh Ewald algorithms, respectively. The system was then simulated for 100 ns.

Post-simulation structural analyses were performed on the WT and mutants using various trajectories focusing at the mutation site, dimer and tetramer interfaces, and protein–ligand affinities.

### Statistical analysis

The calculations of sensitivity and specificity were performed (https://www.medcalc.org/calc/diagnostic_test.php), according to the following parameters: sensitivity = TP/(TP + FN) × 100; specificity = TN/(TN + FP) × 100. The results were expressed as percentage with 95% confidence interval (CI). The G6PD activity of the population was expressed as median ± interquartile range using GraphPad Prism (GraphPad Software, La Jolla, CA, USA).

## Results

### Malaria

**Fig. 2:**
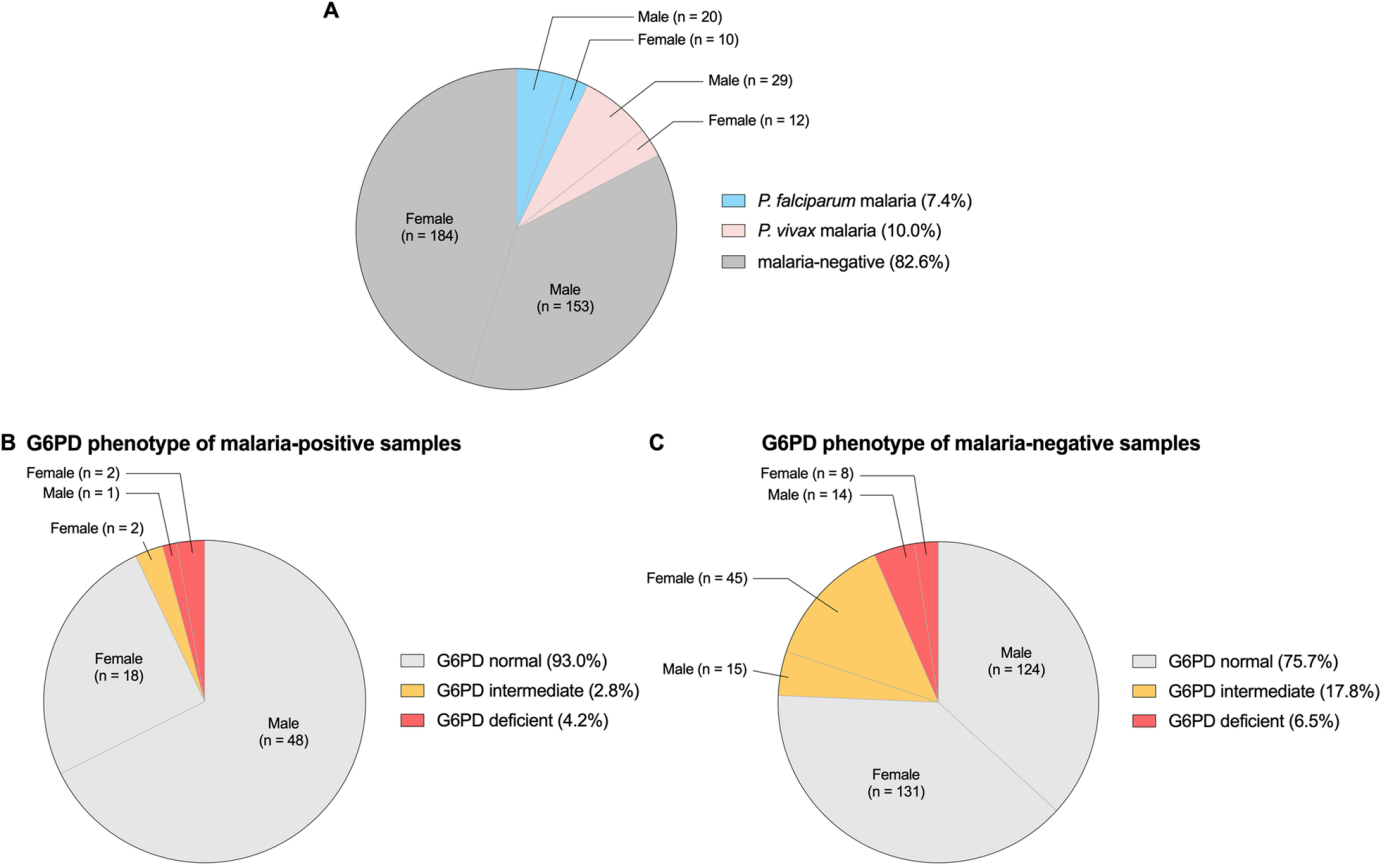
A pie chart depicting **A** the proportion of malaria cases among the studied population and the prevalence of G6PD deficiency in **B** malaria-positive samples and **C** malaria-negative samples

Of the 408 tested subjects, there were 202 male participants and 206 female participants. Malaria was detected in 71 samples with 30 cases (42.30%) of *Plasmodium falciparum* and 41 cases (57.70%) of *P. vivax*, Fig. 2A.

### Prevalence of G6PD deficiency

**Fig. 3:**
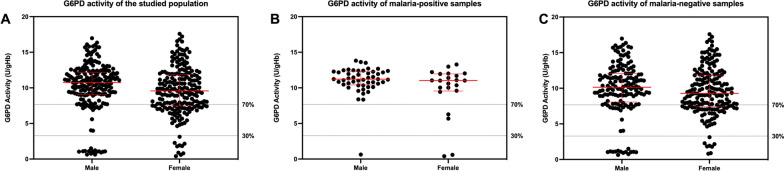
Distribution of G6PD enzyme activity in **A** all participants, **B** malaria-positive samples and **C** malaria-negative samples

Based on WST-8 phenotypic test, the normal median of the studied population was 10.94 ± 2.35 U/gHb. To identify those who will be eligible for primaquine and tafenoquine treatment, G6PD activity of < 30% of the normal median (< 3.28 U/gHb) was defined as G6PD deficient; and G6PD activity between 30 and 70% of the normal median (3.28–7.66 U/gHb) was defined as G6PD intermediate [41–43]. The overall prevalence of G6PD deficiency was 6.13% (25/408) and G6PD intermediate accounted for 15.20% (62/408) of the studied population. In malaria patients, one male and two females were considered G6PD deficient and two females were considered G6PD intermediate (Fig. 2B). In malaria-negative samples, 22 samples (14 males and 8 females) were G6PD deficient and 60 samples (15 males and 45 females) were G6PD intermediate (Fig. 2C). The distribution of G6PD activity among all studied population, malaria-positive, and malaria-negative samples is shown in Fig. 3. The frequency distribution of enzyme activity in male and female participants is depicted in Additional file 1: Fig. S2.

### *G6PD* genotypes

The sensitivity and specificity of the developed HRM assays for detecting 15 *G6PD* mutations were 100% (CI 96.38–100%) and 100% (CI 95.20–100%), respectively. Among 408 samples, mutation detection by HRM assays revealed various *G6PD* mutations, with new genotypes identified.

36.62% (26/71) of malaria patients were found to carry seven *G6PD* genotypes (Table 1). The most common *G6PD* genotype found was a combination of synonymous c.1311C>T and intronic c.1365-13T>C mutations, which accounted for 69.23% (18/26) of detected genotypes. As expected *G6PD* Viangchan, the most common missense variant in the Thai population, was found as compound mutations (c.871 G>A, c.1311C>T, and c.1365-13T>C). The missense *G6PD* Mahidol was also detected as a compound mutation, in combination with synonymous and intronic mutations (c.487G>A, c.1311C>T, and c.1365-13T>C). A new genotype, the double missense mutation *G6PD* Viangchan + Canton (c.871G>A, c.1376G>T, c.1311C>T and c.1365-13T>C), was identified in two females. Other single mutations detected were the missense *G6PD* Valladolid (c.406C>T), a deletion in intron 5 (c.486-34delT) and an intron 11 mutation (c.1365-13T>C).

**Table 1:** *G6PD* genotypes among malaria-positive samples

Genotype	Variant name	N			Frequency (%)
		Male	Female*	Total	
c.406C>T	Valladolid	0	1	1	3.85
c.487G>A, c.1311C>T, c.1365-13T>C	Mahidol	0	1	1	3.85
c.871G>A, c.1311C>T, c.1365-13T>C	Viangchan	1	0	1	3.85
**c.871G**>**A,** **c.1376G**>**T,** **c.1311C**>**T,** **c.1365-13T**>**C**	Viangchan Canton	0	2	2	7.69
c.486-34delT		1	1	2	7.69
c.1365-13T>C		1	0	1	3.85
c.1311C>T, c.1365-13T>C		13	5	18	69.23
Total				26	100

Bold indicates new *G6PD* genotype identified in this study

^*^The zygosity of *G6PD* variants in females was not determined

The frequency of *G6PD* mutations (50.74%) was higher among 337 malaria-negative samples with 171 samples carrying *G6PD* genotypes that were predominantly multiple mutations (Table 2). The highest frequency (95/171, 56%) was observed for the combination of synonymous c.1311C>T and intronic c.1365-13T>C mutations, similar to that found in malaria patients. Notably, variants previously found as single missense mutations were detected along with this combination: *G6PD* Gaohe (c.95A>G, c.1311C>T, and c.1365-13T>C), *G6PD* Valladolid (c.406C>T, c.1311C>T, and c.1365-13T>C), and *G6PD* Chinese-5 (c.1024C>T, c.1311C>T, and c.1365-13T>C). The second most common genotype was a deletion in intron 5, c.486-34delT (26/171, 15%), which was also found as a compound mutation (c.486-34delT, c.1311C>T, and c.1365-13T>C). The third most frequent variant was the compound mutation containing *G6PD* Viangchan (c.871G>A, c.1311C>T, and c.1365-13T>C). Interestingly, HRM assays identified new *G6PD* genotypes in association with *G6PD* Viangchan among malaria-negative samples: *G6PD* Gaohe + Viangchan (c.95A>G, c.871G>A, c.1311C>T, and c.1365-13T>C), *G6PD* Valladolid + Viangchan (c.406C>T, c.871G>A, c.1311C>T, and c.1365-13T>C), and a compound mutation (c.871G>A, c.486-34delT, c.1311C>T, and c.1365-13T>C). Eight single missense mutations were observed:

*G6PD* Canton (c.1376G>T), *G6PD* Mahidol (c.487G>A), *G6PD* Gaohe (c.95A>G), *G6PD* Aures (c.143T>C), *G6PD* Chinese-4 (c.392G>T), *G6PD* Mediterranean (c.563C>T), *G6PD* Chinese-5 (c.1024C>T), and *G6PD* Union (c.1360C>T). An intronic variant (c.1365-13T>C) and a synonymous mutation (c.519C>T) were detected as well. Among malaria-negative samples, 6 samples with impaired G6PD activity were subjected to DNA sequencing and four single nucleotide substitutions were identified; namely, *G6PD* Kaiping (c.1388G>A), *G6PD* Vanua Lava (c.383T>C), *G6PD* Gond (c.477G>C), and a combination of intron 7 mutation (c.771-39C>T), c.1311C>T, and c.1365-13T>C.

**Table 2:** *G6PD* genotypes among malaria-negative samples

Genotype	Variant name	N			Frequency
		Male	Female*	Total	
c.95A>G	Gaohe	0	1	1	0.58%
c.95A>G, c.1311C>T, c.1365-13T>C	Gaohe	0	1	1	0.58%
c.95A>G, c.871G>A, c.1311C>T, c.1365-13T>C	Gaohe, Viangchan	0	1	1	0.58%
c.143T>C	Aures	0	1	1	0.58%
**c.383T**>**C**	**Vanua Lava**	0	1	1	0.58%
c.392G>T	Chinese-4	0	1	1	0.58%
c.406C>T, c.1311C>T, c.1365-13T>C	Valladolid	1	0	1	0.58%
c.406C>T, c.871G>A, c.1311C>T, c.1365-13T>C	Valladolid, Viangchan	0	1	1	0.58%
**c.477G**>**C**	**Gond**	0	1	1	0.58%
c.487G>A	Mahidol	1	1	2	1.17%
c.519C>T		0	1	1	0.58%
c.563C>T	Mediterranean	1	0	1	0.58%
c.871G>A, c.1311C>T, c.1365-13T>C	Viangchan	6	10	16	9.28%
c.871G>A, c.486-34delT, c.1311C>T, c.1365-13T>C	Viangchan	0	1	1	0.58%
c.1024C>T	Chinese-5	0	1	1	0.58%
c.1024C>T, c.1311C>T, c.1365-13T>C	Chinese-5	0	1	1	0.58%
c.1360C>T	Union	1	0	1	0.58%
c.1376G>T	Canton	2	2	4	2.34%
**c.1388G**>**A**	**Kaiping**	1	2	3	1.75%
c.486-34delT		11	15	26	15.20%
c.1365-13T>C		1	2	3	1.75%
c.1311C>T, c.1365-13T>C		35	60	95	55.56%
c.486-34delT, c.1311C>T, c.1365-13T>C		0	6	6	3.51%
**c.771-39C**>**T** c.1311C>T, c.1365-13T>C		0	1	1	0.58%
Total				171	100%

Bold indicates *G6PD* genotypes identified by DNA sequencing

^*^The zygosity of *G6PD* variants in females was not determined

### Phenotype-genotype association analysis

Among the studied population, single missense mutations in hemizygotes and multiple missense mutations in compound heterozygotes gave rise to deficient phenotype with enzyme activity values less than 30% of the normal median (Additional file 1: Fig. S3). In heterozygous females, single missense and synonymous mutations can result in G6PD activities ranging from deficient to normal. A female heterozygous for a synonymous mutation (c.519C>T) showed a deficient phenotype, with enzyme activity of 2.27 U/gHb. Individuals carrying the deletion mutation (c.486-34delT) showed intermediate and normal phenotypes while those carrying the intronic mutation (c.1365-13T>C) showed a normal phenotype, with G6PD activity ranges of 5.06‒15.22 and 10.36‒12.03 U/gHb for deletion and intronic mutations, respectively. Both males and females carrying the synonymous c.1311C>T and intronic c.1365-13T>C mutations showed a wide range of enzyme activity values, ranging from intermediate to normal. The enzyme activity values were comparable between individuals with the combination of c.1311C>T and c.1365-13T>C mutations and those with the compound mutations (c.486-34delT, c.1311C>T, and c.1365-13T>C).

### Biochemical properties and structural stability of G6PD variants

Each G6PD mutation was found to affect the catalytic activity of the enzyme to varied degrees. Among the single missense variants, G6PD Gond and G6PD Valladolid had a minor effect on catalytic activity while G6PD Gaohe, G6PD Viangchan, and G6PD Canton had a considerable effect, with G6PD Canton showing the lowest catalytic activity among others (Table 3). These single variants did not alter binding affinity toward both substrates (G6P and NADP^+^), except for G6PD Gond, G6PD Viangchan, and G6PD Canton. The Canton mutation increased binding affinity toward G6P substrate while G6PD Gond and G6PD Viangchan decreased binding affinity toward NADP^+^ substrate. The double missense mutations (G6PD Gaohe + Viangchan, G6PD Valladolid + Viangchan, and G6PD Canton + Viangchan) resulted in less catalytically active enzymes, compared to the WT and corresponding single mutations. G6PD Canton + Viangchan was the least active enzyme among the double variants with increased binding affinity toward G6P substrate, attributable to the presence of Canton mutation.

**Table 3:** Kinetic parameters of recombinant G6PD variants

Construct	Amino acid change	k_cat_ (s^–1^)	K_m_ G6P (μM)	K_m_ NADP^+^ (μM)
WT	–	293.2 ± 7.0	42.1 ± 3.6	12.9 ± 3.3
Gaohe	His27Arg	96.6 ± 3.6	53.0 ± 6.8	11.0 ± 3.4
Valladolid	Arg136Cys	282.3 ± 4.4	50.1 ± 2.7	8.4 ± 2.6
Gond	Met159Ile	255.5 ± 6.5	64.0 ± 5.3	19.5 ± 6.5
Viangchan	Val298Met	107.7 ± 7.4	58.7 ± 5.0	18.0 ± 4.1
Canton	Arg459Leu	64.8 ± 2.6	25.5 ± 4.1	10.5 ± 3.9
Gaohe + Viangchan	His27Arg + Val298Met	57.0 ± 3.2	47.9 ± 7.4	10.3 ± 2.2
Valladolid + Viangchan	Arg136Cys + Val298Met	140.3 ± 3.0	31.0 ± 2.8	8.2 ± 1.4
Canton + Viangchan	Arg459Leu + Val298Met	22.6 ± 1.2	17.3 ± 3.6	9.6 ± 2.3

The presence of mutations did not alter the secondary structure of G6PD variants, Additional file 1: Fig. S4. Based on the three-dimensional structure, human G6PD is an α-helical protein, containing two domains: β + α domain and a coenzyme binding domain with a classic β-α-β dinucleotide-binding fold [44]. CD spectra of G6PD variants showed two negative peaks at 208 and 222 nm, which are characteristics of the α-helical protein. All G6PD variants showed similar CD absorption spectra to that of the WT enzyme albeit with varying absorption intensities, which could be attributed to changes in flexibility or rigidity of the secondary structure.

**Fig. 4:**
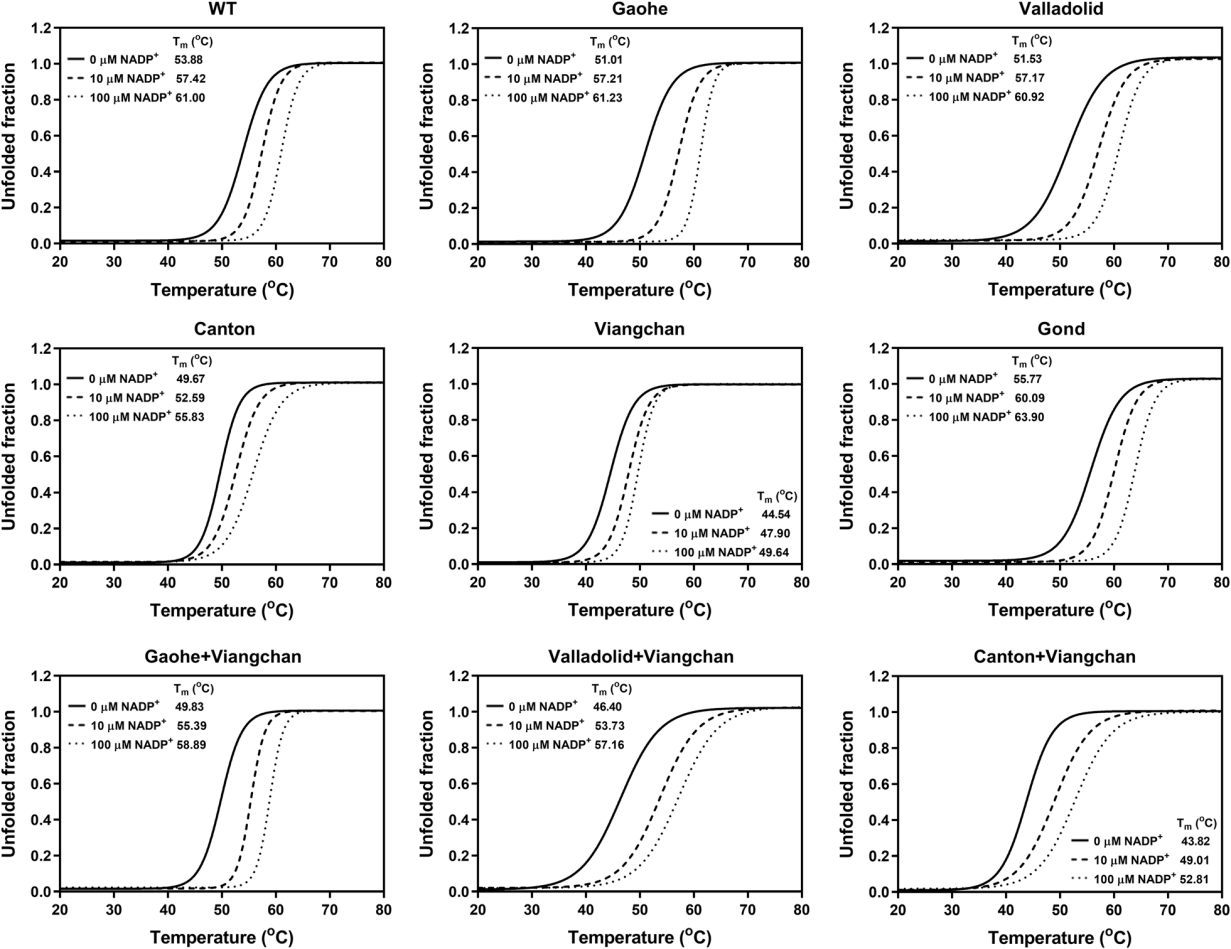
Thermal stability analysis of G6PD variants. The reaction mixtures were heated at temperatures ranging from 20 °C to 80 °C for 20 min, with excitation and emission wavelengths of 465 and 580 nm, respectively. Residual enzyme activity was measured and the melting temperature (*T*_*m*_) was calculated and defined as the temperature at which half of the protein unfolded

The presence of mutations was found to destabilise G6PD protein structures, with different mutations causing varying degrees of structural instability. A second NADP^+^‒binding site is found in the three-dimensional structure of G6PD protein, proximal to the dimer interface, and plays a critical role in structural stabilisation [38]. Herein, structural stability tests were carried out in varying concentrations of NADP^+^. In the thermal shift assay, *T*_*m*_ values of recombinant G6PD proteins in the presence of different concentrations of NADP^+^ are shown in Fig. 4 and Additional file 1: Table S3. Notably, the single missense G6PD Gond showed even a greater structural stability (with higher *T*_*m*_ values) than the WT enzyme, both in the absence and presence of NADP^+^. The Valladolid mutation had the least effect on structural stability when compared to the WT enzyme, whereas the Viangchan variant had the greatest effect, with *T*_*m*_ values of 53.88 °C, 51.53 °C, and 44.54 °C for G6PD WT, G6PD Valladolid, and G6PD Viangchan, respectively. The combination of two missense mutations had additionally reduced structural stability of the protein, with G6PD Canton + Viangchan showing the lowest *T*_*m*_ of 43.82 °C. The presence of NADP^+^ was found to stabilise protein structure in a concentration-dependent manner for all variants.

**Fig. 5:**
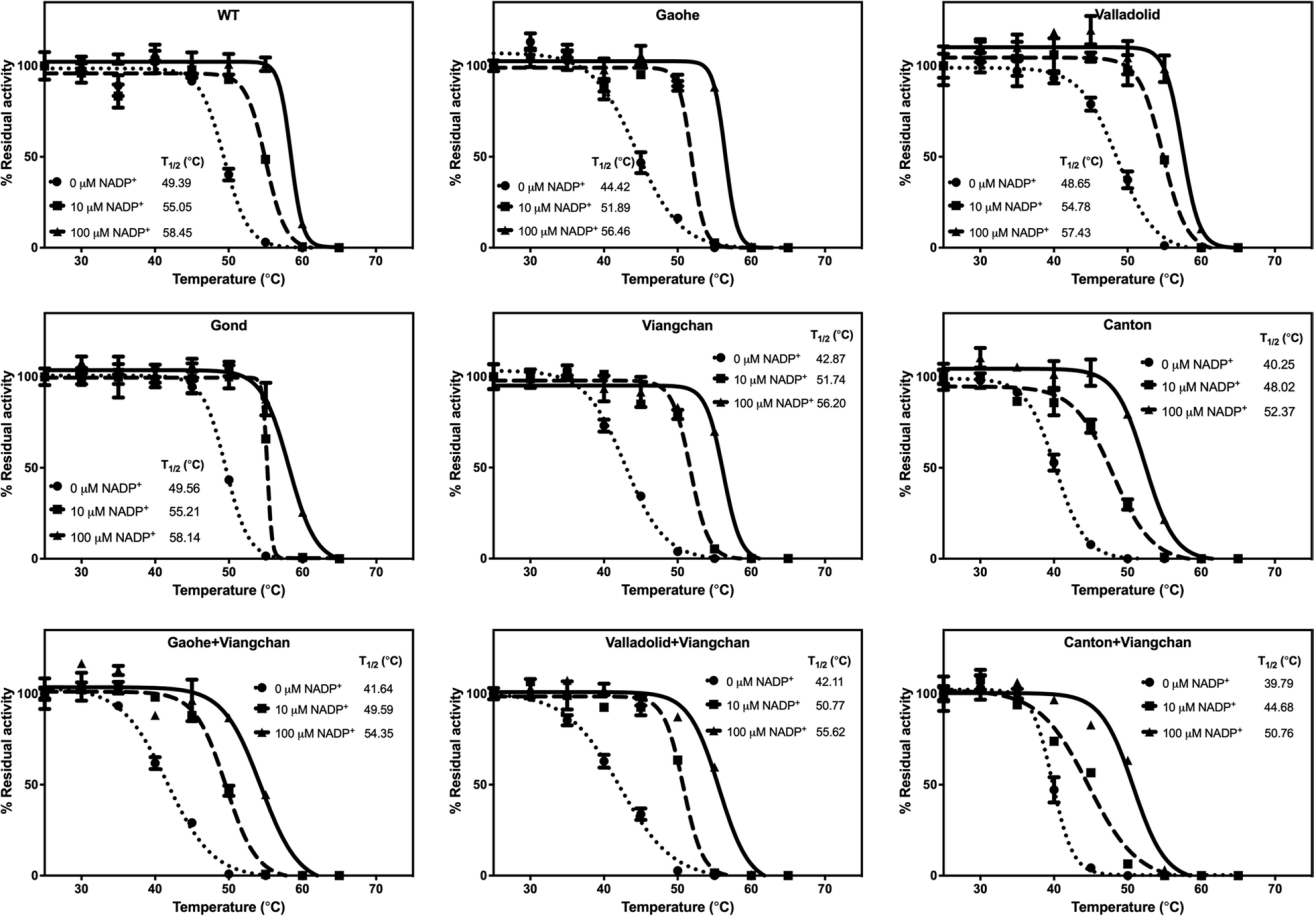
Thermal inactivation analysis of recombinant G6PD variants. Residual enzyme activity was measured after the protein was heated at different temperatures (25 to 65 °C) for 20 min in the presence of various concentrations of NADP^+^ (0, 10 and 100 μM). T_1/2_ is the temperature at which the enzyme loses 50% activity. Error bars represent mean ± SD of triplicate measurements

In agreement with the thermal shift assay, the thermal inactivation test also revealed different destabilising effects among G6PD variants (Fig. 5 and Additional file 1: Table S4). Upon exposure to increasing temperatures, protein denatures and loses its activity. Measurement of residual enzyme activity can be used to assess structural stability, as measured by T_1/2_. The Valladolid mutation had only a small effect on structural stability, showing T_1/2_ value of 48.65 °C which is comparable to that of the WT enzyme (T_1/2_ = 49.39 °C). G6PD Canton was the least stable of the single missense variants, while G6PD Canton + Viangchan was the least stable of the double missense variants, with T_1/2_ values of 40.25 °C and 39.79 °C for G6PD Canton and G6PD Canton + Viangchan, respectively. The combined effect of double mutations on structural instability was also evident in the thermal activity assay.

**Fig. 6:**
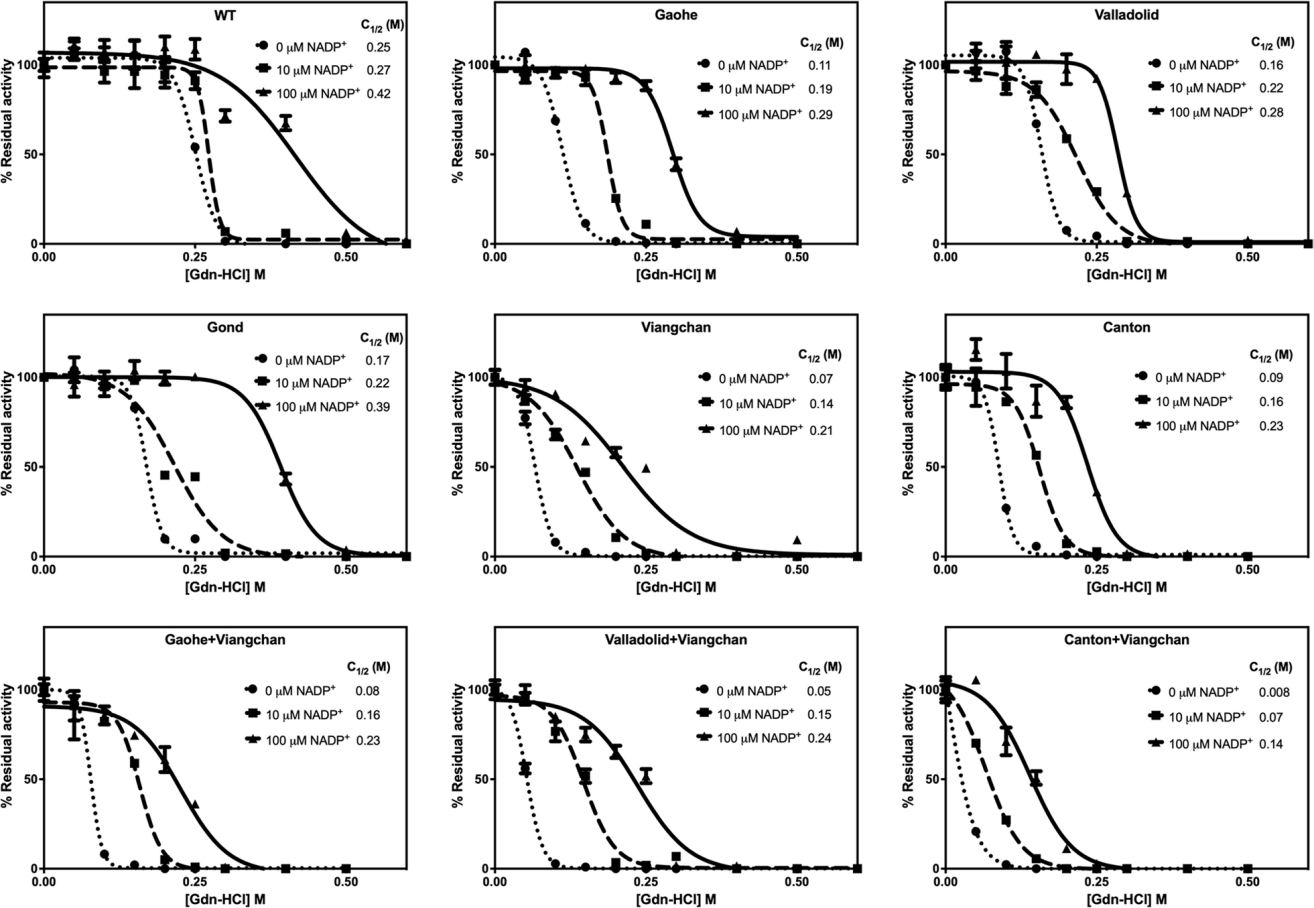
Structural stability analysis of recombinant G6PD variants upon Gdn-HCl treatment. Residual enzymatic activity was measured after incubation with different concentrations of Gdn-HCl (0‒0.5 M) at 37 °C for 2 h in the presence of various concentrations of NADP^+^ (0, 10 and 100 μM). Residual enzyme activity is expressed as a percentage of the activity for the same enzyme incubated in the absence of Gdn-HCl. C_1/2_ is the Gdn-HCl concentration at which the enzyme loses 50% of its activity. Error bars represent mean ± SD of triplicate measurements

Structural stability in the presence of different concentrations of Gdn-HCl, a chemical denaturant, was evaluated (Fig. 6 and Additional file 1: Table S5). Protein structure unfolds upon treatment with increasing concentrations of Gdn-HCl, and the structural stability of G6PD variants can be determined by measuring residual enzyme activity. Protein with greater structural stability, as measured by C_1/2_, is more resistant to Gdn-HCl treatment. The WT enzyme was most resistant to Gdn-HCl with the C_1/2_ of 0.25 M. Among the single variants, G6PD Viangchan and G6PD Gond were the least and the most stable variants upon Gdn-HCl treatment with C_1/2_ values of 0.07 M and 0.17 M, respectively. When compared to the WT enzyme and their respective single mutations, the double variants were more susceptible to chemical denaturation, reflecting less structural stability. G6PD Canton + Viangchan, the least stable G6PD variant studied here, lost 50% of its activity in the presence of as low as 0.008 M of Gdn-HCl.

**Fig. 7:**
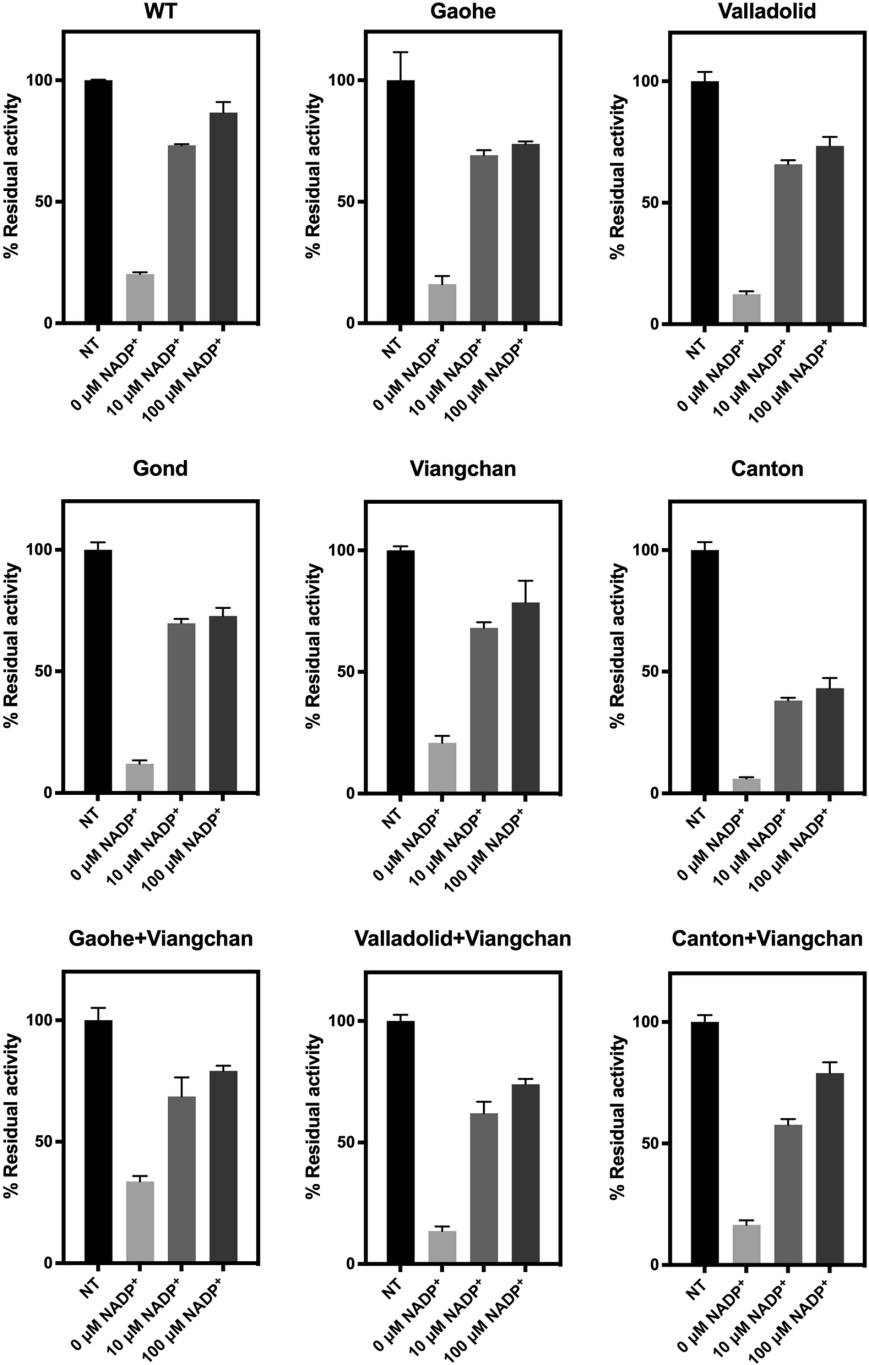
Susceptibility of recombinant G6PD variants to trypsin digestion. Residual enzymatic activity was measured after incubation with 0.5 mg/mL trypsin at 25 °C for 5 min in the presence of various concentrations of NADP^+^ (0, 10 and 100 μM). Residual enzyme activity was expressed as a percentage of the activity for the same enzyme in the absence of trypsin. Error bars represent the mean ± SD of triplicate measurements

Susceptibility to trypsin digestion was also assessed in order to examine the structural stability of G6PD variants (Fig. 7 and Additional file 1: Table S6). G6PD Canton was the most sensitive variant to trypsin digestion in this assay, with a residual activity of 6%, whereas the WT and G6PD Viangchan retained 20% of their activity. The stabilising effect of NADP^+^ was observed for all variants. Similar to other structural stability tests, trypsin digestion indicated that the double variants were less structurally stable than their corresponding single variants.

### Structural analysis of G6PD variants by molecular docking and molecular dynamic simulation

MDS approach was utilised to understand the mechanisms as to how G6P and NADP^+^ influence protein multimerisation and how variants arising from different regions of the protein affect enzyme activity. Mutation-induced structural changes due to single and double missense mutations were critically analysed by calculating the average distance and number of hydrogen bonds made between the mutation site and its neighboring residues (Table 4). The Canton mutation is located close to the dimer interface which induces loss of interhelical interactions between αe (177–190) and αn (455–473) [20]. This mutation is shown to increase the distance between monomeric subunits of the dimer which causes the structure to be loosely packed, characterised by a higher radius of gyration (Rg), thus resulting in reduced enzyme activity (Additional file 1: Tables S7 and 8). The Viangchan mutation demonstrated high fluctuations of amino acid side chains at the substrate and cofactor binding sites, as well as dimer and tetramer interfaces, that led to the instability of the enzyme structure as determined by the root mean square fluctuation and the root-mean-square deviation values (Additional file 1: Table S8). Fluctuation of the amino acid side chains at the catalytic binding pocket disrupts the hydrogen bond interactions between NADP^+^ and Lys171, hence impairing the catalytic activity (Additional file 1: Fig. S5). The Canton + Viangchan mutation demonstrated similar structural changes at the mutation site as the corresponding single variants. This mutation led to deleterious enzyme activity due to loss of hydrogen bond interactions at the NADP^+^ binding sites; and major conformational changes of the amino acids at the ligand binding sites and dimer and tetramer interfaces (Additional file 1: Fig. S5 and Table S8). Despite loss of Asp421–Asp421 interactions at the dimer interface, the dimeric form remained intact through hydrogen bond interaction between Ser418 and Thr423 but this hindered tetramer formation characterised by low tetramer salt bridge SASA (Additional file 1: Table S7).

**Table 4:** A comparison of the intermolecular interactions between the mutation site and neighbouring residues for the WT and variants

Mutation site			Neighbouring residue	Hydrogen bonds between residues (WT)		Hydrogen bonds between residues (Variant)	
Variant	Position	Mutated residue		Distance (Å)	Bonds	Distance (Å)	Bonds
Canton	459	Arg-> Leu (αn 455–473)	Asp 181 (αe 177–190)	2.6	1	3.7	0
Viangchan	291	Val- > Met (αj 281–292)	Cys 294 (αj–αk loop 293–299)	2.3	0	2.4	0
**Canton + Viangchan** **(R459L + V291M)**	**459**	**Arg-> Leu** **(αn 455–473)**	**Asp 181** **(αe 177–190)**	**2.6**	**1**	**3.8**	**0**
	**291**	**Val- > Met** **(αj 281–292)**	**Cys 294** **(αj–αk loop 293–299)**	**2.3**	**0**	**2.4**	**0**
Valladolid	136	Arg- > Cys (βD 136–140)	Asn 122 (αc 115–132)	3.2	5	–	2
			Cys 158 (αd-βE loop 157–164)	3.2		–	
			Gly 131 (αc 115–132)	3.1		–	
			Arg 166 (αd-βE loop 157–164)	3.0		2.1	
			Trp 164 (αd-βE loop 157–164)	2.8		2.5	
Viangchan	291	Val- > Met (αj 281–292)	Cys 294 (αj**–**αk loop 293–299)	2.3	0	2.4	0
**Valladolid + Viangchan** **(R136C + V291M)**	**342**	**Arg- > Cys** **(βD 136–140)**	**Asn 122 (αc 115–132)**	**3.2**	**5**	**–**	**2**
			**Cys 158 (αd-βE loop 157- 164)**	**3.2**		**–**	
			**Gly 131 (αc 115–132)**	**3.1**		**–**	
			**Arg 166 (αd-βE loop 157- 164)**	**3.0**		**1.8**	
			**Trp 164 (αd-βE loop 157- 164)**	**2.8**		**1.9**	
	**291**	**Val- > Met** **(αj 281–292)**	**Cys 294** **(αj–αk loop 293–299)**	**2.3**	**0**	**3.3**	**0**
Gaohe	32	His- > Arg (βA 32–37)	Phe 66 (βB 65–71)	1.8	2	1.9	2
Viangchan	291	Val- > Met (αj 281–292)	Cys 294 (αj–αk loop 293–299)	2.3	0	2.4	0
**Gaohe + Viangchan** **(H32R + V291M)**	**32**	**His- > Arg** **(βA 32–37)**	**Phe 354** **(βJ 353–360)**	**1.8**	**2**	**1.9**	**2**
	**291**	**Val- > Met** **(αj 281–292)**	**Cys 294** **(αj–αk loop 293–299)**	**2.3**	**0**	**2.4**	**1**
Gond	159	Met- > Ile (αd-βE loop 157–164)	Ser 157 (αd 145–158)	2.7	1	3.3	0

Bold indicates double mutations

Mutation in G6PD Valladolid happened at a highly conserved region near the catalytic NADP^+^ binding pocket which disrupts the interactions with αc (115–132) and αd-βE loop (Table 4). This variant is unable to retain the G6P–Lys171 interaction at the substrate binding site but has high occupancy for NADP^+^. In the Valladolid + Viangchan variant, both Lys171–G6P and Lys171-NADP^+^ hydrogen bonds are absent (Additional file 1: Fig. S5). The distance between Met291 and Cys294 shifted by 1.0 Å causing increased distance between βN–βN strands at the dimer interface (Table 4). Loss of interactions with G6P, NADP^+^ and βN–βN strands observed in the Valladolid + Viangchan variant affects the affinity of the ligands.

G6PD Gaohe variant, located close to the catalytic domain, affected the substrate and NADP^+^ binding as the Lys171 amino acid was unable to retain hydrogen bonds with G6P and NADP^+^ (Additional file 1: Fig. S5). High Rg determines that Gaohe is structurally dissimilar to the WT and low SASA of the tetramer salt bridge residues indicates hindered tetramerisation, hence reasoning for its low enzyme activity despite this variant is able to retain important interactions at the dimer interface (Additional file 1: Tables S7 and S8). The double variant G6PD Gaohe + Viangchan recorded lower enzyme activity attributable to the Gaohe variant, due to loss of hydrogen bonds at the substrate and cofactor binding sites (Additional file 1: Fig. S5).

Loss of polar contacts between αd-βE loop and αd helix in the G6PD Gond variant is due to the mutation at residue 159, leading to displacement of the βE-αe loop (residues 170 – 176) (Table 4) and a reduced number of hydrogen bonds at the G6P binding site (Additional file 1: Fig. S5). This loop plays a vital role in directing G6P and NADP^+^ to their respective binding pockets, hence affecting the affinity of both molecules.

Based on the structural analysis of G6PD variants, it is crucial for the G6PD structures to retain the Lys171-G6P and Lys171-c.NADP^+^ hydrogen bonds via the βE-αe loop. It was also evident that high structural integrity at the dimer and tetramer interfaces was important for G6PD structures to express high enzyme activity. Mutation-induced structural changes in the βE-αe loop and the dimer and tetramer interfaces are the underlying reasons for the reduced enzyme activity in these G6PD variants. A summary of structural changes in these variants is provided in Additional file 1: Fig. S6.

## Discussion

Phenotypic testing revealed prevalence of 4.22% and 6.53% among malaria-positive and malaria-negative samples, respectively. Genetic testing revealed *G6PD* mutation frequencies of 36.62% in malaria-positive samples and 50.74% in malaria-negative samples. The findings here are in agreement with previous reports regarding the prevalence of G6PD deficiency in the Thai population [23, 35]. However, with limited sample number, the retrospective nature of this study, the diverse geographic origins of the participants, and the fact that *P. vivax* and *P. falciparum* infections have very different red blood cell preferences, the protective effect of G6PD deficiency against malaria infection cannot be affirmed. According to the 70% cut-off, 7% of malaria-positive samples were ineligible for tafenoquine treatment. It was much higher in the control group, with 24.33% having enzyme activities less than 70% of the normal median. It should be noted that there was no clear consensus about whether 60% or 70% or 80% would be a more appropriate value for a threshold of G6PD enzyme activity. The 70% cut-off used in this study was based on the exclusion criteria for tafenoquine clinical trials, allowing heterozygous females with intermediate enzyme activity who are at risk of haemolysis to be excluded [42, 43]. Setting the threshold too low risks misclassifying patients as G6PD normal and exposing them to drug-induced haemolysis, while setting it too high risks excluding G6PD normal patients from obtaining radical treatment, putting them at risk of relapse and associated morbidity [45].

The combination of synonymous c.1311C>T and intronic c.1365-13T>C mutations was found to be the most frequent *G6PD* genotype in both studied groups. These mutations are common polymorphic markers among Asian populations and are regarded to be of no functional significance because they do not alter the protein sequence. However, previous studies have shown that the double mutations (c.1311C>T and c.1365-13T>C) without *G6PD* mutations in the coding regions were associated with decreased G6PD enzyme activity among Chinese, Thai, Palestinian and Kachin populations [46–49]. Therefore, it was suggested that G6PD deficiency could be caused not only by a single mutation in the exon or exon‒intron boundaries, but also by a haplotype of the *G6PD* gene [48].

Both c.1311C>T and c.1365-13T>C were predicted to have no splicing or deleterious effect on the *G6PD* gene [50–52]. While c.1365-13T>C was found to be benign, the c.1311C>T showed conflicting results and the clinical interpretations of pathogenicity were still uncertain on the ClinVar database (https://www.ncbi.nlm.nih.gov/clinvar/). The combination of c.1311C>T and c.1365-13T>C was found in 2.56% of G6PD-deficient individuals and 15.7% of normal samples in southern China [53], while it was found in 0.38% and 15.4% of the Han Chinese population in G6PD-deficient and G6PD-normal, respectively [54]. The combination of c.1311C>T and c.1365-13T>C resulted in a wide range of G6PD activity in this study, ranging from intermediate to normal and the frequency of these combined polymorphisms was higher in malaria-positive samples. To assess whether this combination has an impact on malaria treatment, further information is required to completely understand the association of c.1311C>T/ c.1365-13T>C and drug-induced haemolysis.

The c.486-34delT resulted in a wide range of G6PD activity (5.06‒15.22 U/gHb). This variant was neutral based on splicing and functional predictions [50–52]. While the c.486-34delT variant was described as benign by many clinical testing groups it was found to be associated with G6PD deficiency in unrelated hemizygotes on the ClinVar database. The variant was also reported to give rise to deficient and normal phenotypes in the Chinese population [34, 53]. Despite the fact that the c.486-34delT variant was linked to enzyme deficiency, there was no indication of haemolytic toxicity, hence no concerns were raised regarding administration of 8-aminoquinolines in people with the c.486-34delT variant.

Although synonymous and intronic mutations do not alter protein sequences, they could have functional effects on gene regulation processes, such as transcription factor binding, transcription, pre-mRNA splicing, mRNA folding and stability, translational initiation, efficiency and accuracy, as well as co-translational protein folding [55]. While growing evidence suggests that synonymous mutations are non-neutral in other genes, the implications of synonymous and intronic mutations on the *G6PD* gene remain largely unknown [55]. Hence, more investigation is needed to elucidate molecular mechanisms underlying G6PD enzyme deficiency caused by synonymous and intronic mutations.

It should be noted that single missense mutations as well as compound mutations of single missense, synonymous and intronic variants frequently gave rise to intermediate to normal enzyme activity, especially in heterozygous females because of individual variation in the patterns of X-chromosome inactivation (lyonisation), which results in variation in the number of circulating normal- and deficient-red blood cells [56]. This could lead to misidentification of G6PD status by commonly used qualitative tests, including the rapid diagnostic tests (RDTs), posing significant haemolytic risks in vulnerable individuals. Therefore, genetic testing can be used as a complement to phenotypic testing, especially in individuals with inconclusive or unexpected phenotypic results, in order to correctly identify those at risk of drug-induced haemolysis.

Previously, *G6PD* Gond was reported in Indian and Arab populations [57–59]. While the Gond mutation (Met159Ile) can result in impaired enzyme activity, the G6PD deficiency in a Saudi male with G6PD Gond was only mild, with 53% activity [57]. In this study, DNA sequencing identified *G6PD* Gond in a heterozygous female with enzyme activity of 6.09 U/gHb (55.67% activity). To our knowledge, this is the first report of *G6PD* Gond in the Thai population. The Met159Ile mutation had only a slight effect on catalytic activity with a small reduction in binding affinity for both substrates. Alteration in binding affinity was attributable to displacement of the βE-αe loop involved in directing G6P and NADP^+^ to their respective binding pockets. The mutation was found to contribute to enzyme deficiency as a result of structural instability. G6PD Gond was structurally less stable than the WT enzyme, showing lower thermal stability as well as greater susceptibility to chemical denaturation and trypsin digestion. Though the effects of the Gond mutation were found to be mild and no evidence on haemolysis was described, cautions should be taken as other G6PD variants with mild deficiency have been associated with haemolytic toxicity [60, 61].

Interestingly, three new double missense mutations were identified by HRM assays in compound heterozygous females with G6PD deficiency, including *G6PD* Gaohe + Viangchan (c.95A>G, c.871G>A, c.1311C>T, and c.1365-13T>C), *G6PD* Valladolid + Viangchan (c.406C>T, c.871G>A, c.1311C>T, and c.1365-13T>C) and *G6PD* Viangchan + Canton (c.871G>A, c.1376G>T, c.1311C>T, and c.1365-13T>C). Individual mutation of Gaohe, Valladolid, Viangchan, and Canton resulted in varying degrees of enzyme deficiency, with G6PD Canton having the lowest enzyme activity caused by impaired dimerisation. All four mutations destabilised protein structure, mainly due to conformational changes at βE-αe loop, dimer and tetramer interfaces, resulting in decreased thermal stability and increased susceptibility to chemical denaturation and trypsin treatment when compared to the WT enzyme. The Viangchan and Canton mutations have a major impact on structural stability, attributable to disruption of oligomeric interactions. When compared to the WT and their corresponding single mutations, the combination of two missense mutations resulted in less catalytically active enzymes with remarkably lower structural stability, contributing to severe enzyme deficiency. As expected from the individual mutations, the double mutant Viangchan + Canton was the least active variant based on biochemical properties, structural stability analysis and molecular dynamic simulation. The findings here indicate that structural instability plays an important role in contributing to enzyme deficiency caused by Gaohe, Valladolid, Viangchan, and Canton mutations. All double missense mutations, including those reported here, exhibited a severe enzyme deficient phenotype, making them ineligible for 8-aminoquinoline prescription due to high haemolytic risk [36].

## Conclusions

Molecular analysis revealed that the Thai population has distinct characteristic profiles of *G6PD* mutations with a high frequency of synonymous and intronic mutations, resulting in intermediate to normal enzyme activity. While the impact of synonymous and intronic mutations is still uncertain, more investigation is required to study molecular mechanisms underlying G6PD deficiency as well as the association with haemolytic toxicity. Heterozygous females carrying single missense mutations as well as compound mutations of single missense and synonymous/intronic mutations showed a wide range of enzyme activity. This suggests that genetic testing might be required as a complement to phenotypic analysis to correctly identify those at risk of drug-induced haemolysis in the studied population. The single and double missense mutations identified here resulted in different degrees of enzyme deficiency. The combined effect of double missense mutations was observed, with double missense mutations resulting in less catalytically enzyme than their corresponding single missense mutations. Among the missense mutations addressed here, structural instability was revealed to have a significant contribution in causing enzyme deficiency.

### Supplementary Information

**Additional file 1: Figure S1.** Primers used in (A) multiplex HRM for the detection of 15 *G6PD* mutations and (B) *G6PD* gene sequencing. **Table S1**. Primers used in multiplex HRM assays. **Table S2.** Primers used for site-directed mutagenesis. **Figure S2.** The frequency distribution of G6PD enzyme activity in (A) males and (B) females. **Figure S3.** Box plot of G6PD activity for each variant among (A) malaria-positive males, (B) malaria-positive females, (C) malaria-negative males and (D) malaria-negative females. **Figure S4**. Secondary structure analysis of G6PD variants by circular dichroism. **Table S3.** Melting temperature (*T*_*m*_) values of recombinant G6PD proteins by thermal shift assay. Mutations were ranked in order of stability, from most stable to least stable. **Table S4.** Thermal inactivation of G6PD variants as reported by T_1/2_. Mutations were ranked in order of stability, from most stable to least stable. **Table S5.** Stability of G6PD variants in the presence of Gdn-HCl as reported by C_1/2_. Mutations were ranked in order of stability, from most stable to least stable. **Table S6.** Susceptibility of G6PD variants to trypsin digestion. Mutations were ranked in order of stability, from most stable to least stable. **Table S7.** Structural characteristics of the dimer and tetramer interfaces (t = 100 ns). **Table S8.** Average values of the trajectory analyses performed on the WT and variants. **Figure S5.** Ligand binding pocket occupancy heatmap indicating the presence (orange) and absence (turquoise) of hydrogen bonds (t = 100 ns). **Figure S6.** Superimposition and structural deviations of the simulated variants against the WT (red) at the mutation site, dimer and tetramer interfaces (t = 100 ns). (A) Gaohe, (B) Valladolid, (C) Canton, (D) Viangchan, (E) Gond, (F) Gaohe + Viangchan, (G) Valladolid + Viangchan, and (H) Canton + Viangchan.

## Data Availability

All data analysed during the study are included in this published article.

## Abbreviations

CD: Circular dichroism
CI: Confidence interval
G6P: Glucose-6-phosphate
G6PD: Glucose-6-phosphate dehydrogenase
Gdn-HCl: Guanidine hydrochloride
Hb: Haemoglobin
HRM: High-resolution melting
MDS: Molecular dynamic simulation
NADPH: Reduced nicotinamide adenine dinucleotide phosphate
Rg: Radius of gyration
WT: Wild type
WHO: World Health Organization
WST-8: Water-soluble tetrazolium salts

## References

[1] WHO (2022). World malaria report 2022.

[2] WHO (2021). Global technical strategy for malaria 2016–2030.

[3] Beutler E (1989). Glucose-6-phosphate dehydrogenase: new perspectives. Blood.

[4] Motulsky AG (1989). Metabolic polymorphisms and the role of infectious diseases in human evolution. Hum Biol.

[5] Eggleston LV, Krebs HA (1974). Regulation of the pentose phosphate cycle. Biochem J.

[6] Gaetani GD, Parker JC, Kirkman HN (1974). Intracellular restraint: a new basis for the limitation in response to oxidative stress in human erythrocytes containing low-activity variants of glucose-6-phosphate dehydrogenase. Proc Natl Acad Sci USA.

[7] Alving AS, Carson PE, Flanagan CL, Ickes CE (1956). Enzymatic deficiency in primaquine-sensitive erythrocytes. Science.

[8] Pamba A, Richardson ND, Carter N, Duparc S, Premji Z, Tiono AB (2012). Clinical spectrum and severity of hemolytic anemia in glucose 6-phosphate dehydrogenase-deficient children receiving dapsone. Blood.

[9] Motulsky AG, Campbell-Kraut JM, Blumberg BS (1960). Population genetic of glucose-6-phosphate dehydrogenase deficiency of the red cell. Proceedings of the Conference on genetic polymorphisms and geographic variations in disease.

[10] Allison AC (1960). Glucose-6-phosphate dehydrogenase deficiency in red blood cells of East Africans. Nature.

[11] Siniscalco M, Bernini L, Filippi G, Latte B, Meera Khan P, Piomelli S (1966). Population genetics of haemoglobin variants, thalassaemia and glucose-6-phosphate dehydrogenase deficiency, with particular reference to the malaria hypothesis. Bull World Health Organ.

[12] Bienzle U, Ayeni O, Lucas AO, Luzzatto L (1972). Glucose-6-phosphate dehydrogenase and malaria: greater resistance of females heterozygous for enzyme deficiency and of males with non-deficient variant. Lancet.

[13] Guindo A, Fairhurst RM, Doumbo OK, Wellems TE, Diallo DA (2007). X-linked G6PD deficiency protects hemizygous males but not heterozygous females against severe malaria. PLoS Med.

[14] Mbanefo EC, Ahmed AM, Titouna A, Elmaraezy A, Trang NT, Phuoc Long N (2017). Association of glucose-6-phosphate dehydrogenase deficiency and malaria: a systematic review and meta-analysis. Sci Rep.

[15] Awab GR, Aaram F, Jamornthanyawat N, Suwannasin K, Pagornrat W, Watson JA (2021). Protective effect of Mediterranean-type glucose-6-phosphate dehydrogenase deficiency against *Plasmodium vivax* malaria. Elife.

[16] Morris SA, Crews KR, Hayden RT, Takemoto CM, Yang W, Baker DK (2022). Incorporating G6PD genotyping to identify patients with G6PD deficiency. Pharmacogenet Genomics.

[17] Uyoga S, Ndila CM, Macharia AW, Nyutu G, Shah S, Peshu N (2015). Glucose-6-phosphate dehydrogenase deficiency and the risk of malaria and other diseases in children in Kenya: a case-control and a cohort study. Lancet Haematol.

[18] Luzzatto L, Ally M, Notaro R (2016). Glucose-6-phosphate dehydrogenase deficiency. Blood.

[19] WHO (2022). Meeting report of the technical consultation to review the classification of glucose-6-phosphate dehydrogenase (G6PD).

[20] Minucci A, Moradkhani K, Hwang MJ, Zuppi C, Giardina B, Capoluongo E (2012). Glucose-6-phosphate dehydrogenase (G6PD) mutations database: review of the "old" and update of the new mutations. Blood Cells Mol Dis.

[21] Geck RC, Powell NR, Dunham MJ (2023). Functional interpretation, cataloging, and analysis of 1,341 glucose-6-phosphate dehydrogenase variants. Am J Hum Genet.

[22] Tanphaichitr VS, Pung-amritt P, Yodthong S, Soongswang J, Mahasandana C, Suvatte V (1995). Glucose-6-phosphate dehydrogenase deficiency in the newborn: its prevalence and relation to neonatal jaundice. Southeast Asian J Trop Med Public Health.

[23] Nuchprayoon I, Sanpavat S, Nuchprayoon S (2002). Glucose-6-phosphate dehydrogenase (G6PD) mutations in Thailand: G6PD Viangchan (871G>A) is the most common deficiency variant in the Thai population. Hum Mutat.

[24] Laosombat V, Sattayasevana B, Janejindamai W, Viprakasit V, Shirakawa T, Nishiyama K (2005). Molecular heterogeneity of glucose-6-phosphate dehydrogenase (G6PD) variants in the south of Thailand and identification of a novel variant (G6PD Songklanagarind). Blood Cells Mol Dis.

[25] Ninokata A, Kimura R, Samakkarn U, Settheetham-Ishida W, Ishida T (2006). Coexistence of five G6PD variants indicates ethnic complexity of Phuket islanders, Southern Thailand. J Hum Genet.

[26] Charoenkwan P, Tantiprabha W, Sirichotiyakul S, Phusua A, Sanguansermsri T (2014). Prevalence and molecular characterization of glucose-6-phosphate dehydrogenase deficiency in northern Thailand. Southeast Asian J Trop Med Public Health.

[27] Nantakomol D, Paul R, Palasuwan A, Day NP, White NJ, Imwong M (2013). Evaluation of the phenotypic test and genetic analysis in the detection of glucose-6-phosphate dehydrogenase deficiency. Malar J.

[28] Boonyuen U, Chamchoy K, Swangsri T, Saralamba N, Day NP, Imwong M (2016). Detailed functional analysis of two clinical glucose-6-phosphate dehydrogenase (G6PD) variants, G6PD_Viangchan_ and G6PD_Viangchan+Mahidol_: decreased stability and catalytic efficiency contribute to the clinical phenotype. Mol Genet Metab.

[29] Sudsumrit S, Chamchoy K, Songdej D, Adisakwattana P, Krudsood S, Adams ER (2022). Genotype-phenotype association and biochemical analyses of glucose-6-phosphate dehydrogenase variants: Implications for the hemolytic risk of using 8-aminoquinolines for radical cure. Front Pharmacol.

[30] Chamchoy K, Praoparotai A, Pakparnich P, Sudsumrit S, Swangsri T, Chamnanchanunt S (2021). The integrity and stability of specimens under different storage conditions for glucose-6-phosphate dehydrogenase deficiency screening using WST-8. Acta Trop.

[31] Snounou G, Viriyakosol S, Zhu XP, Jarra W, Pinheiro L, do Rosario VE (1993). High sensitivity of detection of human malaria parasites by the use of nested polymerase chain reaction. Mol Biochem Parasitol.

[32] Boonyuen U, Songdej D, Tanyaratsrisakul S, Phuanukoonnon S, Chamchoy K, Praoparotai A (2021). Glucose-6-phosphate dehydrogenase mutations in malaria endemic area of Thailand by multiplexed high-resolution melting curve analysis. Malar J.

[33] Chen Y, Xiu W, Dong Y, Wang J, Zhao H, Su Y (2018). Mutation of glucose-6-phosphate dehydrogenase deficiency in Chinese Han children in eastern Fujian. Medicine (Baltimore).

[34] Shen S, Xiong Q, Cai W, Hu R, Zhou B, Hu X (2022). Molecular heterogeneity of glucose-6-phosphate dehydrogenase deficiency in neonates in Wuhan: description of four novel variants. Front Genet.

[35] Chamchoy K, Sudsumrit S, Wongwigkan J, Petmitr S, Songdej D, Adams ER (2023). Molecular characterization of G6PD mutations identifies new mutations and a high frequency of intronic variants in Thai females. PLoS ONE.

[36] Pakparnich P, Sudsumrit S, Imwong M, Suteewong T, Chamchoy K, Pakotiprapha D (2021). Combined effects of double mutations on catalytic activity and structural stability contribute to clinical manifestations of glucose-6-phosphate dehydrogenase deficiency. Sci Rep.

[37] Bradford MM (1976). A rapid and sensitive method for the quantitation of microgram quantities of protein utilizing the principle of protein-dye binding. Anal Biochem.

[38] Kotaka M, Gover S, Vandeputte-Rutten L, Au SW, Lam VM, Adams MJ (2005). Structural studies of glucose-6-phosphate and NADP^+^ binding to human glucose-6-phosphate dehydrogenase. Acta Crystallogr D Biol Crystallogr.

[39] Pronk S, Pall S, Schulz R, Larsson P, Bjelkmar P, Apostolov R (2013). GROMACS 4.5: a high-throughput and highly parallel open source molecular simulation toolkit. Bioinformatics.

[40] Malde AK, Zuo L, Breeze M, Stroet M, Poger D, Nair PC (2011). An automated force field topology builder (ATB) and repository: Version 1.0. J Chem Theory Comput.

[41] WHO (2022). Test for glucose-6-phosphate dehydrogenase activity: target product profiles.

[42] Llanos-Cuentas A, Lacerda MVG, Hien TT, Velez ID, Namaik-Larp C, Chu CS (2019). Tafenoquine versus primaquine to prevent relapse of *Plasmodium vivax* malaria. N Engl J Med.

[43] Lacerda MVG, Llanos-Cuentas A, Krudsood S, Lon C, Saunders DL, Mohammed R (2019). Single-dose tafenoquine to prevent relapse of *Plasmodium vivax* malaria. N Engl J Med.

[44] Au SW, Gover S, Lam VM, Adams MJ (2000). Human glucose-6-phosphate dehydrogenase: the crystal structure reveals a structural NADP(^+^) molecule and provides insights into enzyme deficiency. Structure.

[45] Pfeffer DA, Ley B, Howes RE, Adu P, Alam MS, Bansil P, Boum Y, Brito M, Charoenkwan P, Clements A (2020). Quantification of glucose-6-phosphate dehydrogenase activity by spectrophotometry: a systematic review and meta-analysis. PLoS Med.

[46] Jiang W, Yu G, Liu P, Geng Q, Chen L, Lin Q (2006). Structure and function of glucose-6-phosphate dehydrogenase-deficient variants in Chinese population. Hum Genet.

[47] Thedsawad A, Wanachiwanawin W, Taka O, Hantaweepant C (2022). Cut-off values for diagnosis of G6PD deficiency by flow cytometry in Thai population. Ann Hematol.

[48] Sirdah MM, Shubair ME, Al-Kahlout MS, Al-Tayeb JM, Prchal JT, N Scott Reading (2017). Possible association of 3' UTR +357 A>G, IVS11-nt 93 T>C, c.1311 C>T polymorphism with G6PD deficiency. Hematology.

[49] Li Q, Yang F, Liu R, Luo L, Yang Y, Zhang L (2015). Prevalence and molecular characterization of glucose-6-phosphate dehydrogenase deficiency at the China-Myanmar border. PLoS ONE.

[50] Rentzsch P, Schubach M, Shendure J, Kircher M (2021). CADD-Splice-improving genome-wide variant effect prediction using deep learning-derived splice scores. Genome Med.

[51] Jaganathan K, Kyriazopoulou Panagiotopoulou S, McRae JF, Darbandi SF, Knowles D, Li YI (2019). Predicting splicing from primary sequence with deep learning. Cell.

[52] Cheng J, Nguyen TYD, Cygan KJ, Celik MH, Fairbrother WG, Avsec Z, Gagneur J (2019). MMSplice: modular modeling improves the predictions of genetic variant effects on splicing. Genome Biol.

[53] Lin F, Lou ZY, Xing SY, Zhang L, Yang LY (2018). The gene spectrum of glucose-6-phosphate dehydrogenase (G6PD) deficiency in Guangdong province. China Gene.

[54] Yan JB, Xu HP, Xiong C, Ren ZR, Tian GL, Zeng F, Huang SZ (2010). Rapid and reliable detection of glucose-6-phosphate dehydrogenase (G6PD) gene mutations in Han Chinese using high-resolution melting analysis. J Mol Diagn.

[55] Shen X, Song S, Li C, Zhang J (2022). Synonymous mutations in representative yeast genes are mostly strongly non-neutral. Nature.

[56] Harper PS (2011). Mary Lyon and the hypothesis of random X chromosome inactivation. Hum Genet.

[57] Faiyaz-Ul-Haque M, Zaidi SH, Hasanato RM, Al-Abdullatif A, Cluntun A, Teresita G (2010). Genetics of glucose-6-phosphate dehydrogenase deficiency in Saudi patients. Clin Genet.

[58] Malik S, Zaied R, Syed N, Jithesh P, Al-Shafai M (2021). Seven novel glucose-6-phosphate dehydrogenase (G6PD) deficiency variants identified in the Qatari population. Hum Genomics.

[59] Sarkar S, Biswas NK, Dey B, Mukhopadhyay D, Majumder PP (2010). A large, systematic molecular-genetic study of G6PD in Indian populations identifies a new non-synonymous variant and supports recent positive selection. Infect Genet Evol.

[60] Chu CS, Bancone G, Nosten F, White NJ, Luzzatto L (2018). Primaquine-induced haemolysis in females heterozygous for G6PD deficiency. Malar J.

[61] Chu CS, Bancone G, Soe NL, Carrara VI, Gornsawun G, Nosten F (2019). The impact of using primaquine without prior G6PD testing: a case series describing the obstacles to the medical management of haemolysis. Wellcome Open Res.

