# Supplementary material for: Genetic analysis and molecular basis of G6PD deficiency among malaria patients in Thailand: implications for safe use of 8-aminoquinolines
Source: Malar J. 2024 Feb 2;23:38. doi: 10.1186/s12936-024-04864-8 (PMC10835850; doi:10.1186/s12936-024-04864-8)
Supplement: Supplementary file 1 — Additional file 1: Figure S1. Primers used in (A) multiplex HRM for the detection of 15 G6PD mutations and (B) G6PD gene sequencing. Table S1. Primers used in multiplex HRM assays. Table S2. Primers used for site-directed mutagenesis. Figure S2. The frequency distribution of G6PD enzyme activity in (A) males and (B) females. Figure S3. Box plot of G6PD activity for each variant among (A) malaria-positive males, (B) malaria-positive females, (C) malaria-negative males and (D) malaria-negative females. Figure S4. Secondary structure analysis of G6PD variants by circular dichroism. Table S3. Melting temperature (Tm) values of recombinant G6PD proteins by thermal shift assay. Mutations were ranked in order of stability, from most stable to least stable. Table S4. Thermal inactivation of G6PD variants as reported by T1/2. Mutations were ranked in order of stability, from most stable to least stable. Table S5. Stability of G6PD variants in the presence of Gdn-HCl as reported by C1/2. Mutations were ranked in order of stability, from most stable to least stable. Table S6. Susceptibility of G6PD variants to trypsin digestion. Mutations were ranked in order of stability, from most stable to least stable. Table S7. Structural characteristics of the dimer and tetramer interfaces (t = 100 ns). Table S8. Average values of the trajectory analyses performed on the WT and variants. Figure S5. Ligand binding pocket occupancy heatmap indicating the presence (orange) and absence (turquoise) of hydrogen bonds (t = 100 ns). Figure S6. Superimposition and structural deviations of the simulated variants against the WT (red) at the mutation site, dimer and tetramer interfaces (t = 100 ns). (A) Gaohe, (B) Valladolid, (C) Canton, (D) Viangchan, (E) Gond, (F) Gaohe + Viangchan, (G) Valladolid + Viangchan, and (H) Canton + Viangchan. [file 12936_2024_4864_MOESM1_ESM.docx]

**Additional file**

**Prevalence, genetic analysis and molecular basis of G6PD deficiency among malaria patients in Thailand**

Usa Boonyuen^1,*^, Beatriz Aira C Jacob^1^, Jutamas Wongwigkan^1^, Kamonwan Chamchoy^2^, Natsamon Singha-art^1^, Natnicha Pengsuk^1^, Duantida Songdej^3^, Emily R Adams^4^, Thomas Edwards^4^, Supat Chamnanchanunt^5^, Syazwani Itri binti Amran^6^, Nurriza Ab Latif^6^, Naveen Eugene Louis^6^, Shamini Chandran^6^

^1^ Department of Molecular Tropical Medicine and Genetics, Faculty of Tropical Medicine, Mahidol University, Bangkok, Thailand

^2^ Princess Srisavangavadhana College of Medicine, Chulabhorn Royal Academy, Bangkok, Thailand

^3^ Department of Pediatrics, Faculty of Medicine Ramathibodi Hospital, Mahidol University, Bangkok, Thailand

^4^ Centre for Drugs and Diagnostics Research, Liverpool School of Tropical Medicine, Liverpool, UK

^5^ Department of Clinical Tropical Medicine, Faculty of Tropical Medicine, Mahidol University, Bangkok 10400 Thailand

^6^ Department of Bioscience, Faculty of Science, Universiti Teknologi Malaysia (UTM), Johor Bahru, Malaysia

**Corresponding author**:

Usa Boonyuen, Department of Molecular Tropical Medicine and Genetics, Faculty of Tropical Medicine, Mahidol University, Bangkok, Thailand

Email: [usa.boo@mahidol.ac.th](mailto:usa.boo@mahidol.ac.th), [usa.boo@mahidol.edu](mailto:usa.boo@mahidol.edu)


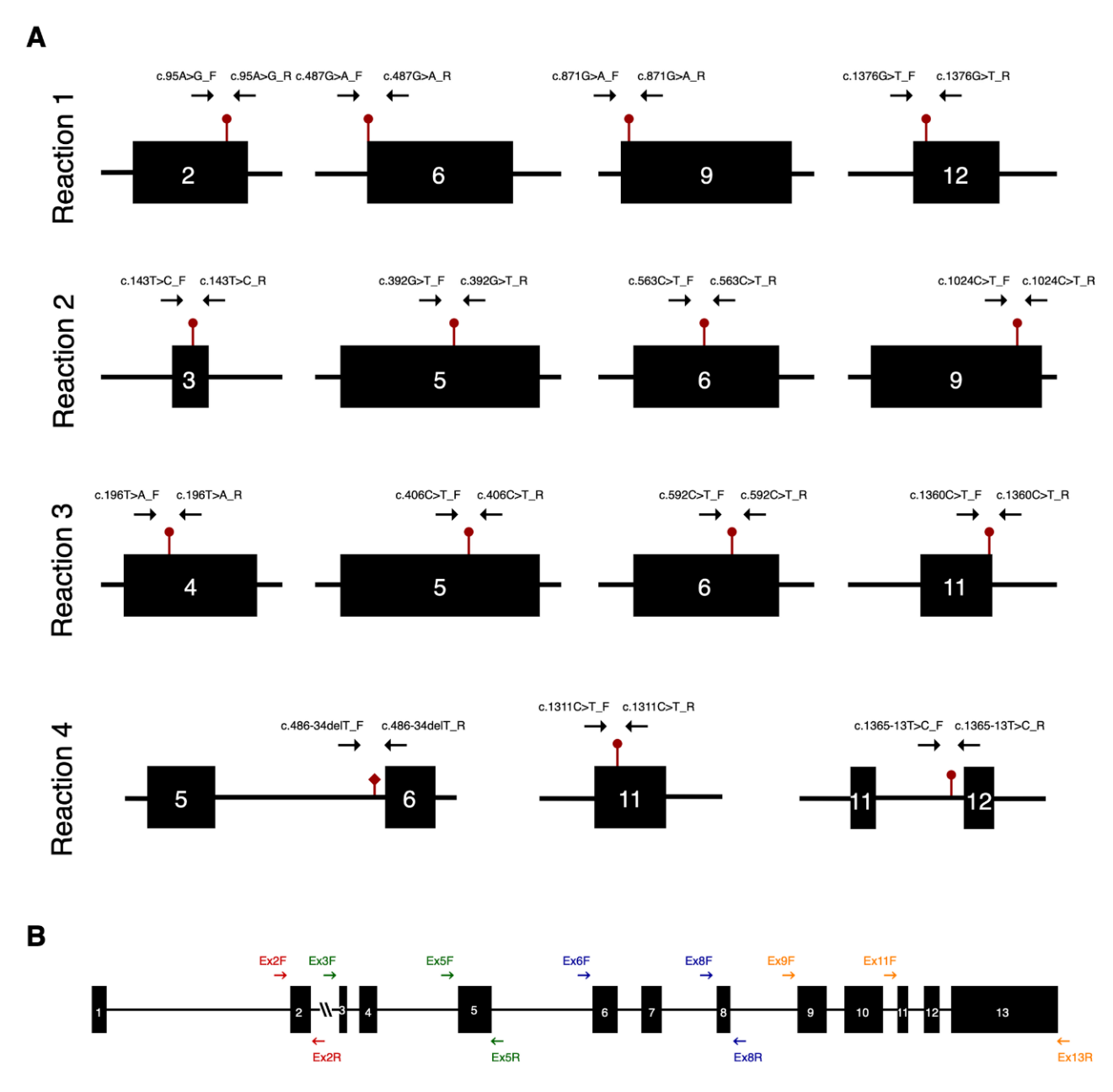


**Figure S1.** Primers used in (A) multiplex HRM for the detection of 15 *G6PD* mutations and (B) *G6PD* gene sequencing.

**Table S1**. Primers used in multiplex HRM assays.

| **Set** | **Variant (Nucleotide change)** | **Primer** | **Primer sequence (5’ to 3’)** | **Primer concentration (nM)** | **Product T_m_**  **(^o^C)** | **Product size (bp)** |
| --- | --- | --- | --- | --- | --- | --- |
| 1 | Gaohe  (c.95A>G) | Forward | TTCCATCAGTCGGATACACG | 600 | 81.16 | 100 |
|  |  | Reverse | AGGCATGGAGCAGGCACTTC |  |  |  |
|  | Mahidol  (c.487G>A) | Forward | TCCGGGCTCCCAGCAGAA | 400 | 84.93 | 87 |
|  |  | Reverse | GGTTGGACAGCCGGTCA |  |  |  |
|  | Viangchan  (c.871G>A) | Forward | GGCTTTCTCTCAGGTCAAGA | 600 | 78.39 | 66 |
|  |  | Reverse | CCCAGGACCACATTGTTGGC |  |  |  |
|  | Canton  (c.1376G>T) | Forward | CCTCAGCGACGAGCTCCT | 600 | 83.74 | 99 |
|  |  | Reverse | CTGCCATAAATATAGGGGATGG |  |  |  |
| 2 | Aures  (c.143T>C) | Forward | GACCTGGCCAAGAAGAAGAC | 400 | 88.43 | 152 |
|  |  | Reverse | AAGGCCATCCCGGAACAGCC |  |  |  |
|  | Chinese-4  (c.392G>T) | Forward | CATGAATGCCCTCCACCTGGT | 200 | 85.46 | 87 |
|  |  | Reverse | TTCTTGGTGACGGCCTCGTA |  |  |  |
|  | Mediterranean (c.563C>T) | Forward | CGGCTGTCCAACCACATCTT | 400 | 82.41 | 87 |
|  |  | Reverse | GTTCTGCACCATCTCCTTGC |  |  |  |
|  | Chinese-5  (c.1024C>T) | Forward | CACTTTTGCAGCCGTCGTCT | 400 | 83.44 | 99 |
|  |  | Reverse | CACACAGGGCATGCCCAGTT |  |  |  |
| 3 | Songklanagarind  (c.196T>A) | Forward | CCTTCTGCCCGAAAACACCA | 400 | 83.76 | 84 |
|  |  | Reverse | AAGGGCTCACTCTGTTTGCG |  |  |  |
|  | Valladolid  (c.406C>T) | Forward | CCTGGGGTCACAGGCCAACT | 400 | 84.64 | 93 |
|  |  | Reverse | CTCATGCAGGACTCGTGAAT |  |  |  |
|  | Coimbra  (c.592C>T) | Forward | CCGTGAGGACCAGATCTACT | 400 | 81.88 | 78 |
|  |  | Reverse | CCCCACCTCAGCACCATG |  |  |  |
|  | Union  (c.1360C>T) | Forward | GAGCCAGATGCACTTCGTGT | 200 | 87.96 | 127 |
|  |  | Reverse | GAGGGGACATAGTATGGCTT |  |  |  |
| 4 | Intron 5 deletion  (c.486-34delT) | Forward | CCTCACTCCCCGAAGAGGGGTC | 800 | 82.32 | 64 |
|  |  | Reverse | TTCCAGCCTCTGCTGGGAGC |  |  |  |
|  | Silent mutation  (c.1311C>T) | Forward | CGTGAAGCTCCCTGACGCCTAT | 200 | 85.26 | 93 |
|  |  | Reverse | CCGGCAGCTGGGCCTCAC |  |  |  |
|  | Intron 11 mutation  (c.1365-13T>C) | Forward | CCGGCCTCCCAAGCCATACC | 400 | 83.64 | 87 |
|  |  | Reverse | CTCAATCTGGTGCAGCAGTGG |  |  |  |

**Table S2.** Primers used for site-directed mutagenesis.

| **Primer** | **Sequence (5’ to 3’)** |
| --- | --- |
| Gaohe_F | GTCGGATACAGGCATATTCATCA |
| Gaohe_R | TGATGAATATGCGTGTATCCGAC |
| Vallodolid_F | ACAGGCCAACTGCCTCTTCTA |
| Vallodolid_R | TAGAAGAGGCAGTTGGCCTGT |
| Gond_F | GTCCTGCATCAGCCAGATA |
| Gond_R | TATCTGGCTGATGCAGGAC |
| Viangchan_F | GATGAGAAGGTCAAGATGTTGAAATGCATC |
| Viangchan_R | GATGCATTTCAACATCTTGACCTTCTCATC |
| Canton_F | GACGAGCTCCTTGAGGCCTGG |
| Canton_R | CCAGGCCTCAAGGAGCTCGTC |


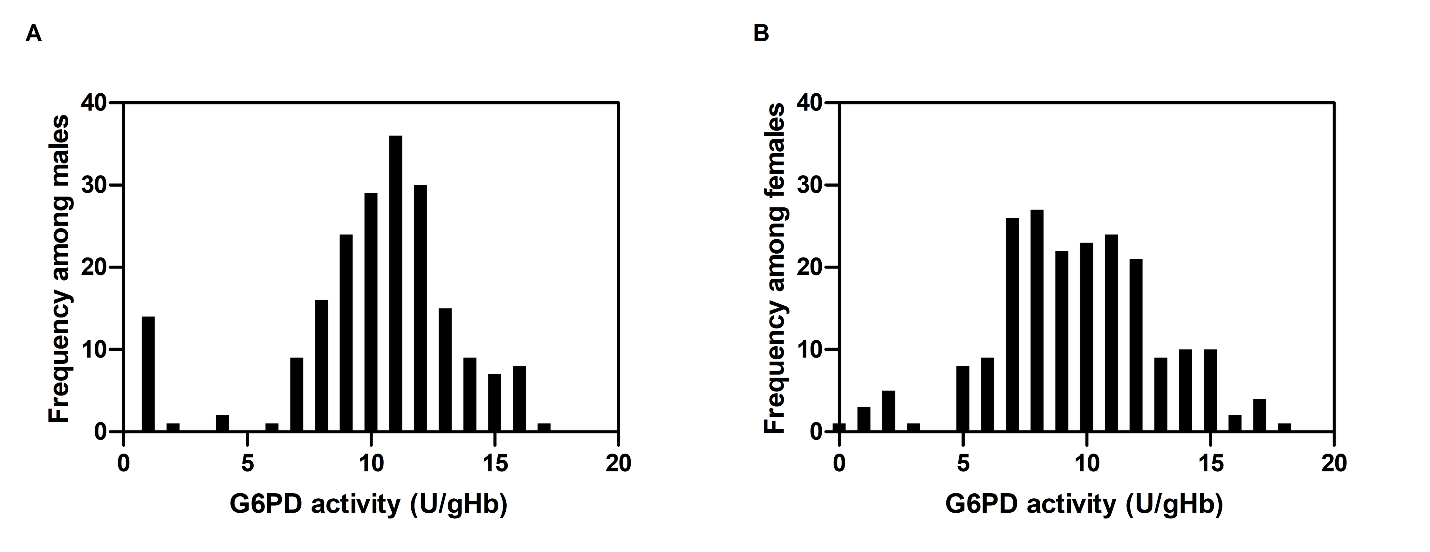


**Figure S2.** The frequency distribution of G6PD enzyme activity in (A) males and (B) females.


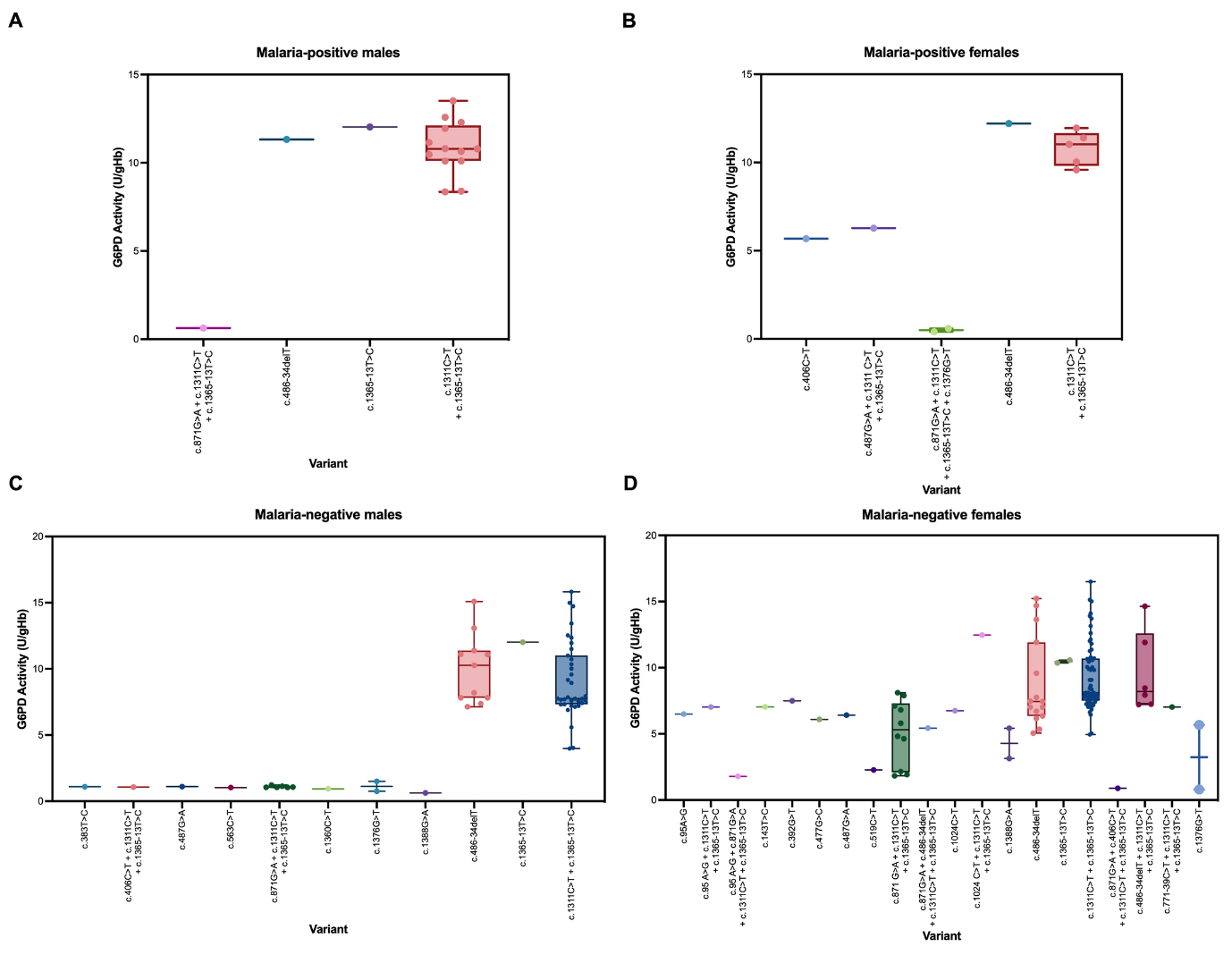


**Figure S3.** Box plot of G6PD activity for each variant among (A) malaria-positive males, (B) malaria-positive females, (C) malaria-negative males and (D) malaria-negative females.


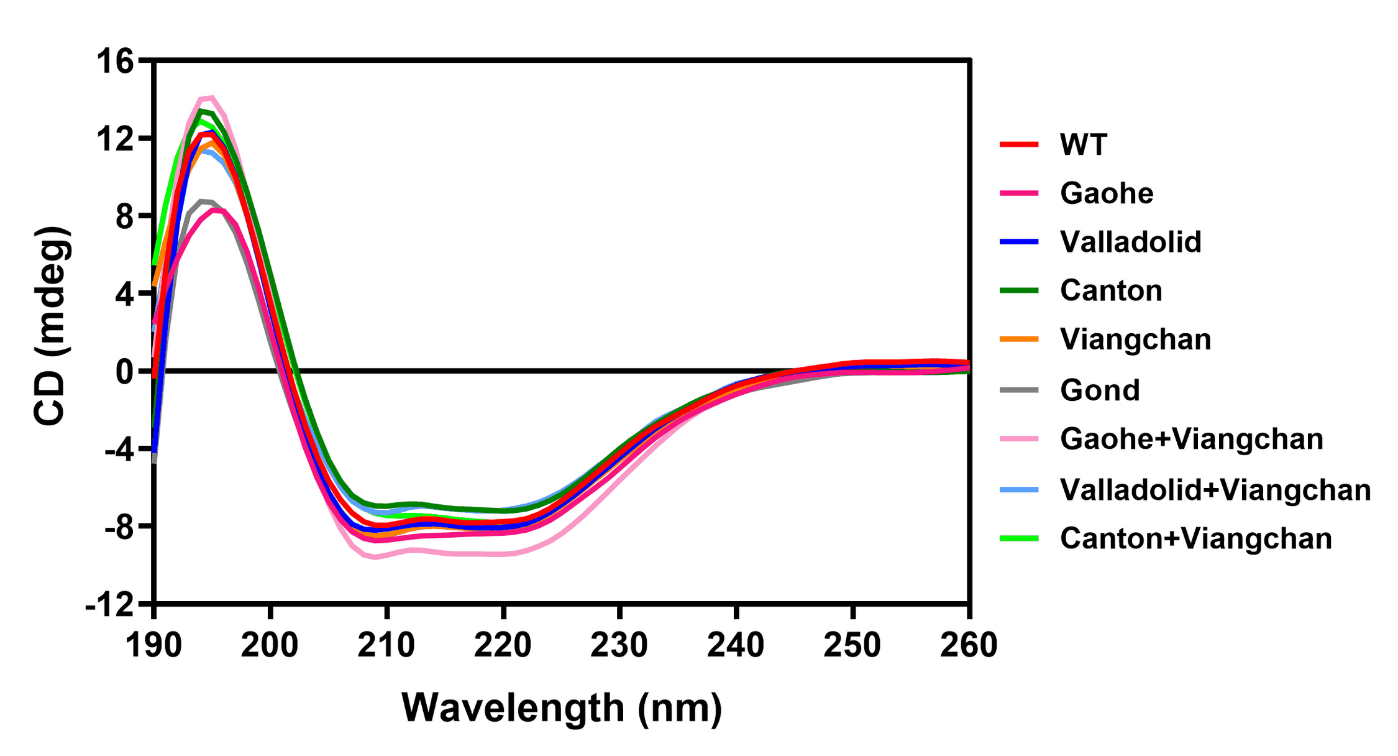


**Figure S4**. Secondary structure analysis of G6PD variants by circular dichroism.

**Table S3.** Melting temperature (*T_m_*) values of recombinant G6PD proteins by thermal shift assay. Mutations were ranked in order of stability, from most stable to least stable.

| **Construct** | **T_m_ (°C)** | | |
| --- | --- | --- | --- |
|  | **0 µM NADP^+^** | **10 µM NADP^+^** | **100 µM NADP^+^** |
| WT | 53.88 | 57.42 | 61.00 |
| Gond | 55.77 | 60.09 | 63.90 |
| Valladolid | 51.53 | 57.17 | 60.92 |
| Gaohe | 51.01 | 57.21 | 61.23 |
| Gaohe+Viangchan | 49.83 | 55.39 | 58.89 |
| Canton | 49.67 | 52.59 | 55.83 |
| Valladolid+Viangchan | 46.40 | 53.73 | 57.16 |
| Viangchan | 44.54 | 47.9 | 49.64 |
| Canton+Viangchan | 43.82 | 49.01 | 52.81 |

**Table S4**. Thermal inactivation of G6PD variants as reported by T_1/2_. Mutations were ranked in order of stability, from most stable to least stable.

| **Variant** | **T_1/2_ (°C)** | | |
| --- | --- | --- | --- |
|  | **0 μM NADP^+^** | **10 μM NADP^+^** | **100 μM NADP^+^** |
| WT | 49.39 | 55.05 | 58.45 |
| Gond | 49.56 | 55.21 | 58.14 |
| Valladolid | 48.65 | 54.78 | 57.43 |
| Gaohe | 44.42 | 51.89 | 56.46 |
| Viangchan | 42.87 | 51.74 | 56.20 |
| Valladolid+Viangchan | 42.11 | 50.77 | 55.62 |
| Gaohe+Viangchan | 41.64 | 49.59 | 54.35 |
| Canton | 40.25 | 48.02 | 52.37 |
| Canton+Viangchan | 39.79 | 44.68 | 50.76 |

**Table S5**. Stability of G6PD variants in the presence of Gdn-HCl as reported by C_1/2_. Mutations were ranked in order of stability, from most stable to least stable.

| **Variant** | **C_1/2_ (M)** | | |
| --- | --- | --- | --- |
|  | **0 μM NADP^+^** | **10 μM NADP^+^** | **100 μM NADP^+^** |
| WT | 0.25 | 0.27 | 0.42 |
| Gond | 0.17 | 0.22 | 0.39 |
| Valladolid | 0.16 | 0.22 | 0.28 |
| Gaohe | 0.11 | 0.19 | 0.29 |
| Canton | 0.09 | 0.16 | 0.23 |
| Gaohe+Viangchan | 0.08 | 0.16 | 0.23 |
| Viangchan | 0.07 | 0.14 | 0.21 |
| Valladolid+Viangchan | 0.05 | 0.15 | 0.24 |
| Canton+Viangchan | 0.008 | 0.07 | 0.14 |

**Table S6**. Susceptibility of G6PD variants to trypsin digestion. Mutations were ranked in order of stability, from most stable to least stable.

| **Variant** | **% Residual activity** | | | |
| --- | --- | --- | --- | --- |
|  | **NT** | **0 μM NADP^+^** | **10 μM NADP^+^** | **100 μM NADP^+^** |
| WT | 100.00 ± 0.24 | 20.14 ± 0.73 | 73.20 ± 0.50 | 86.71 ± 4.30 |
| Gaohe+Viangchan | 100.00 ± 5.09 | 33.61 ± 2.32 | 68.63 ± 7.90 | 79.14 ± 2.13 |
| Viangchan | 100.00 ± 1.73 | 20.93 ± 2.83 | 68.11 ± 2.30 | 78.57 ± 8.91 |
| Canton+Viangchan | 100.00 ± 2.82 | 16.53 ± 1.86 | 57.69 ± 2.36 | 78.94 ± 4.48 |
| Gaohe | 100.00 ± 11.59 | 16.12 ± 3.30 | 69.16 ± 2.09 | 73.86 ± 0.94 |
| Valladolid+Viangchan | 100.00 ± 2.56 | 13.55 ± 1.90 | 62.01 ± 4.76 | 73.96 ± 2.20 |
| Valladolid | 100.00 ± 3.84 | 12.38 ± 1.18 | 65.82 ± 1.69 | 73.35 ± 3.78 |
| Gond | 100.00 ± 3.13 | 12.02 ± 1.39 | 69.75 ± 1.81 | 72.64 ± 3.31 |
| Canton | 100.00 ± 3.33 | 6.05 ± 0.61 | 38.13 ± 1.09 | 43.15 ± 4.20 |

Note: NT is non-treatment

**Table S7**. Structural characteristics of the dimer and tetramer interfaces (t = 100 ns).

|  | Dimer | | | | | | Tetramer |
| --- | --- | --- | --- | --- | --- | --- | --- |
| Protein | Hydrogen bonds (βN – βN) | | | βN - βN distance (Å) | Salt bridges | | SASA of tetramer salt bridge residues (nm^2^) |
|  | Asp 421 - Asp 421 | Glu 419 - Thr 423 | Ser 418 - Thr 423 |  | Glu 206 - Lys 407 | Glu 419 - Arg 427 |  |
| WT | + | + |  | 1.9 | + | - | 20.50 |
| R459L | - | - | - | 2.8 | + | - | 20.33 |
| V291M | + | + | - | 2.3 | + | - | 20.68 |
| **R459L +V291M** | **-** | **-** | **+** | **1.6** | **+** | **-** | **19.37** |
| R136C | - | - | - | 3.4 | + | + | 20.18 |
| V291M | + | + | - | 2.3 | + | - | 20.68 |
| **R136C +V291M** | **-** | **-** | **-** | **5.6** | **+** | **-** | **21.15** |
| H32R | + | - | - | 2.1 | + | + | 20.07 |
| V291M | + | + | - | 2.3 | + | - | 20.68 |
| **H32R + V291M** | **+** | **+** | **-** | **1.8** | **+** | **-** | **20.82** |
| M159I | + | + | - | 2.1 | + | + | 20.64 |

**Table S8**. Average values of the trajectory analyses performed on the WT and variants.

|  | MD parameters | | | | | | | | |
| --- | --- | --- | --- | --- | --- | --- | --- | --- | --- |
| G6PD | RMSD | Rg | Hbond | SASA | RMSF | | | | |
|  |  |  |  |  | c.NADP binding site | G6P binding site | s.NADP binding site | Dimer Interface | Tetramer Interface |
| WT | 0.40 | 3.63 | 774.48 | 20.50 | 0.17 | 0.17 | 0.21 | 0.21 | 0.22 |
| Canton | 0.42 | 3.73 | 787.88 | 20.33 | 0.17 | 0.15 | 0.18 | 0.20 | 0.19 |
| Viangchan | 0.33 | 3.67 | 782.89 | 20.68 | 0.25 | 0.22 | 0.29 | 0.29 | 0.34 |
| Canton + Viangchan | 0.38 | 3.68 | 774.63 | 19.37 | 0.15 | 0.12 | 0.13 | 0.15 | 0.13 |
| Valladolid | 0.36 | 3.70 | 790.00 | 20.18 | 0.16 | 0.11 | 0.15 | 0.15 | 0.15 |
| Valladolid + Viangchan | 0.37 | 3.68 | 774 | 20.87 | 0.16 | 0.13 | 0.15 | 0.16 | 0.18 |
| Gaohe | 0.41 | 3.72 | 772.58 | 20.07 | 0.20 | 0.16 | 0.21 | 0.22 | 0.17 |
| Gaohe + Viangchan | 0.29 | 3.67 | 788.31 | 20.82 | 0.17 | 0.13 | 0.15 | 0.18 | 0.16 |
| Gond | 0.37 | 3.67 | 782.12 | 20.64 | 0.16 | 0.11 | 0.15 | 0.15 | 0.12 |


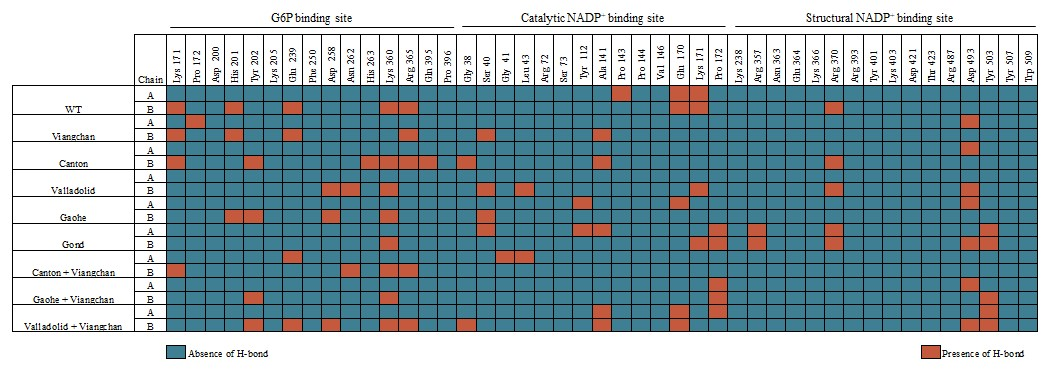


**Figure S5**. Ligand binding pocket occupancy heatmap indicating the presence (orange) and absence (turquoise) of hydrogen bonds (t = 100 ns).


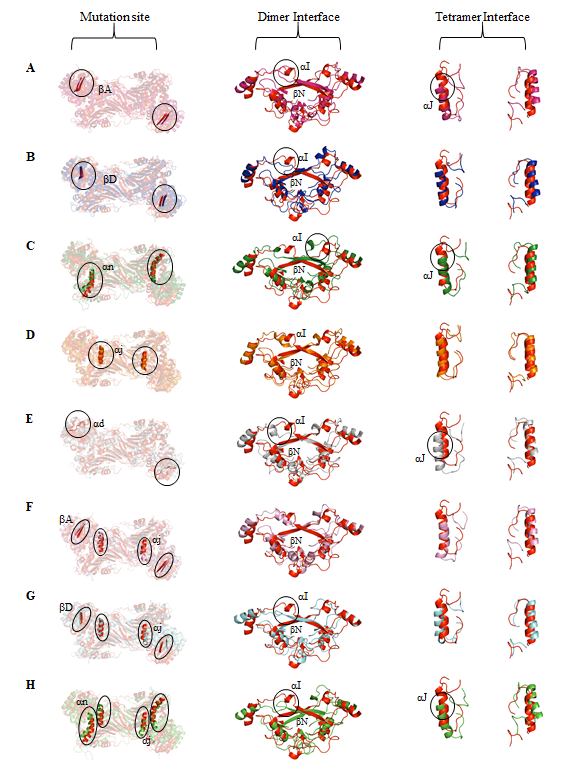


**Figure S6**. Superimposition and structural deviations of the simulated variants against the WT (red) at the mutation site, dimer and tetramer interfaces (t= 100 ns). (A) Gaohe, (B) Valladolid, (C) Canton, (D) Viangchan, (E) Gond, (F) Gaohe + Viangchan, (G) Valladolid + Viangchan, and (H) Canton + Viangchan.
